# Yolk@Wrinkled-double shell smart nanoreactors: new platforms for mineralization of pharmaceutical wastewater

**DOI:** 10.3389/fchem.2023.1211503

**Published:** 2023-06-06

**Authors:** Masoud Habibi Zare, Arjomand Mehrabani-Zeinabad

**Affiliations:** Department of Chemical Engineering, Isfahan University of Technology, Isfahan, Iran

**Keywords:** structural nanoreactor, heterojunction, visible light irradiation, yolk@shell structure, smart nano particles

## Abstract

Nanomaterials with “yolk and shell” “structure” can be considered as “nanoreactors” that have significant potential for application in catalysis. Especially in terms of electrochemical energy storage and conversion, the nanoelectrode has a large specific surface area with a unique yolk@shell structure, which can reduce the volume change of the electrode during the charging and discharging process and fast ion/electron transfer channels. The adsorption of products and the improvement of conversion reaction efficiency can greatly improve the stability, speed and cycle performance of the electrode, and it is a kind of ideal electrode material. In this research, heterojunction nanoreactors (FZT Y@WDS) Fe_3_O_4_@ZrO_2-X_@TiO_2-X_ were firstly synthesized based on the solvothermal combined hard-template process, partial etching and calcination. The response surface method was used to determine the performance of the FZT Y@WDS heterojunction nanoreactors and the effects of four process factors: naproxen concentration (NAP), solution pH, the amount of charged photocatalyst, and the irradiation time for photocatalytic degradation of NAP under visible light irradiation. To maximize the photocatalytic activity, the parameters of the loaded catalyst, the pH of the reaction medium, the initial concentration of NAP, and the irradiation time were set to 0.5 g/L, 3, 10 mg/L, and 60 min, respectively, resulting in complete removal of NAP and the optimum amount was calculated to be 0.5 g/L, 5.246, 14.092 mg/L, and 57.362 min, respectively. Considering the promising photocatalytic activity of FZT Y@WDS under visible light and the separation performance of the nanocomposite, we proposed this photocatalyst as an alternative solution for the treatment of pharmaceutical wastewater.

## Introduction

Pharmaceutical pollution, which has been recognized as one of the major threats to the environment in recent decades, has put people in a critical phase that requires immediate changes and improvements (Kumari and Kumar, 2022). Considering the wide prevalence of COVID-19 disease and the prescription of nonsteroidal drugs for this type of patients, it is predicted that in the near future the effect of this type of drugs will be clearly visible in wastewater (Yousefifard et al., 2020; Zhao et al., 2020; Wong et al., 2021). For this reason, the wastewater from naproxen (NAP) synthesis was selected as the purifying agent for this research work. NAP is a non-steroidal drug and is used to treat many diseases, including rheumatic joint swelling, joint disease, acute gout, and early painful menstruation (Todd and Clissold, 1990). When comparing advanced oxidation methods for the treatment of pharmaceutical wastewater, photocatalytic technology stands out due to low cost, its high efficiency, catalyst structure suitable for any type of wastewater, and stability of catalysts in terms of temperature and corrosion resistance to various wastewater sources (Nivetha et al., 2022). From the catalytic point of view, nanoscale catalysts exhibit enhanced catalytic activity and stability, which have unique and essential properties for chemical reactions. Therefore, nanoreactors have several advantages over conventional reactors, including remove unwanted products, the ability to perform chemical reactions in parallel, and increase catalytic performance due to the large surface-to-volume ratio (Zhang et al., 2022). For this reason, researchers have made great efforts to understand and study nanoreactors, particularly the relationship between catalytic performance and nanoarchitecture (Wu et al., 2022). It has been found that newly developed nanocatalysts for energy conversion have better performance than conventional catalysts, and Yolk@shell catalysts are the best (Hu et al., 2022). Yolk@shell NPs (Y@SNs) or “nanorattles” have attracted much attention due to their unique structure and improved performance in a wide range of applications such as nanomedicine, environmental remediation, conversion and energy storage, and catalysis (Andikaey et al., 2022; Jayan et al., 2022). Catalysts must have special properties, such as even in harsh environments, stability, high catalytic activity and selectivity, stability, reusability, long life. The yolk@shell structure can satisfy all of these requirements since the core of the Y@SN structure is normally the catalytic active site, but the shell protects it during catalysis in order to prevent decay (Ye et al., 2021). In addition, the permeable shell of Y@SN allows sufficient diffusion of reactants into and out of the metal core, preventing agglomeration and sintering of the metal core (Li et al., 2016). There is no doubt that Y@SNs are superior to metal NPs and core-shell NPs as long-term, low-cost catalysts because they overcome numerous serious drawbacks of conventional catalysts (Wang et al., 2017). Hollow structure nanomaterials containing voids in a discrete shell have attracted much attention in the fields of catalysis, energy storage and conversion due to the special properties determined by their unique structure, including large surface area, accessible void, and tunable shell-pore structure (Chieffi et al., 2014; Wang et al., 2016; Lin et al., 2018; Tian et al., 2019a; Gao et al., 2019; Teng et al., 2019; Bi et al., 2020a; Yang et al., 2022). It is believed that the shell of these hollow nanoreactors, which often have an enriched porous structure and a large surface area, can promote high loading of active species (Lee et al., 2014). By encapsulating the active species in the shell, hollow nanoreactors can protect them from sintering, leaching and aggregation even under harsh reaction conditions (Tian et al., 2019b). In addition, hollow nanoreactors can provide molecular sieving capability to realize size-selective and toxin-resistant catalysis (Prieto et al., 2016). Y@SNs of various shapes, sizes, and structures can be readily fabricated by a variety of methods, including Kirkendall effect, selective etching, galvanic exchange, hard template, bottle transport, soft template (or bottom-up methods), Ostwald ripening, or a combination of the above methods (Purbia and Paria, 2015). A large amount of research has been devoted to the development of methods for synthesizing substances, because shape, size, and structure all play important roles in catalytic performance (Purbia and Paria, 2015). When exposed to chemicals, products, or byproducts under experimental conditions, dispersed active nanocores tend to aggregate and lose their catalytic activity. Therefore, the Y@SN shell acts as a protective layer that effectively prevents damage and allows the transfer of reactants and products from the active sites. Finally, the most important function of the yolk@shell nanostructures is to prevent the accumulation and inactivation of active cores (Ray and Pal, 2017). Due to the many advantages of Y@SNs as catalysts for chemical reactions, any research in this field can lead to scientific progress (Purbia and Paria, 2015). Generally, Yolk@shell structures are prepared by the template method (Wu and Xu, 2010). The removal of hard templates often involves etching and high-temperature calcination in air or with controlled chemicals (Wu and Xu, 2010). Changing the etching time or calcination temperature can slightly change the composition and structure of NPs (Liu et al., 2018). The Y@SN structure has also been extensively studied for its ability to utilize unlimited solar energy in addition to generating and converting clean energy (Fahrenbruch and Bube, 2012). A yolk@shell structure traps the irradiated photons and increases the active area of the catalyst, which in turn, together with better utilization of the irradiated light, leads to a reduction in the energy band gaps of the semiconductor materials and their activity in the visible range, which in turn enables a more environmentally friendly process (Li et al., 2019). For many years, scientists and researchers have been searching for catalytic materials that naturally develop their activity after being exposed to visible light (Chen et al., 2010).

Bi_2_WO_6_/Bi_2_S_3_/MoS_2_ spherical ternary composites showed photocatalytic reduction capacity against light, with Cr(VI) reduction rate up to 100% in 75 min (Ren et al., 2020). Mesoporous TiO_2_/Ti_3_C_2_ composites significantly showed the performance of light absorption, separation and transfer of light-induced charge carriers, so that the photocatalytic degradation performance of methyl orange by the mesoporous TiO_2_/Ti_3_C_2_ composite with optimum Ti_3_C_2_ content (3 wt%) reached 99.6% within 40 min. Obtaining. In addition, the optimized mesoporous TiO_2_/Ti_3_C_2_ composite also exhibited an excellent photocatalytic H_2_ production rate of 218.85 μmol.g^–1^. h^–1^, corresponding to 5.6 times activity compared to pure mesoporous TiO_2_ NPs (Li et al., 2022) In another research, an Ag/Ag_3_PO_4_/Ti_3_C_2_ hybrid with 3 wt% Ti_3_C_2_ content achieved the highest MO degradation efficiency and chromium (VI) reduction efficiency after 1 hour irradiation, which were 1.72 and 1.46 times higher than the results obtained with pure Ag_3_PO_4_ NPs, respectively (Sun et al., 2021) As a new 2D metal carbide, nitride or carbonitride, MXene possesses excellent metal conductivity, high charge carrier mobility and ordered band structure, which can be used as a cocatalyst in photocatalytic material systems to improve photocatalytic properties (Zhou et al., 2023).

In this research work, FZT Y@WDS NPs with yolk@shell architecture and S-scheme charge transfer mechanism were synthesized by a seven-step synthesis method. The structure of the smart nanoreactors with yolk@shell architecture was fabricated by removing the silicon oxide (SiO_2_) layer by a partial etching process using caustic soda in air. The size of the cavity between the yolk and the shell can be easily designed by adjusting the thickness of the sacrificial silicon oxide (SiO_2_) layer. In order to design a simple synthesis route for the smart nanoreactors designed with yolk@shell, iron oxide (Fe_3_O_4_) was chosen as the core, and in order to generate magnetic properties, it was also necessary to provide the NPs with the largest photon harvesting area due to the high energy band gap of zirconium oxide (ZrO_2_), so it was chosen as the first shell. On the other hand, it created a resistant and stable property in harsh environments and prevented the recombination of electron-hole pairs for this nanoreactor. Finally, titanium oxide (TiO_2_) showed a very favorable photocatalytic property and was therefore investigated as the second shell in this structure. The smart nanoreactors with FZT Y@WDS architecture synthesized by calcination, solvothermal, and etching methods were characterized by FESEM, XRD, EDS, XPS, BET, TEM, PZC, electrochemical impedance spectroscopy, Matt-Schottky, DRS, polarization, Tafel, CV and UPS analyzes. One of the most important methods for fabricating smart porous nanoreactors is hard-templating of porous SiO_2_, which can be used for separation/adsorption and energy storage. Atomically ordered microstructure closely related to electrical/thermal conductivity and corrosion resistance is a desirable structure closely related to the size and disorder of its microstructure in smart structural nanoreactors. The chemical nature of the precursor and the heat treatment applied usually determine the microstructure of the structural smart nanoreactors. For the first time, the influence of spatial confinement on microstructural evolution in monolithic mesoporous SiO_2_ is investigated using an advanced evaluation approach for wide-angle X-ray scattering data. Based on SEM and N_2_ physisorption measurements, hard-templates are characterized by the presence of precursors exclusively in mesopores. To determine the performance of the smart nanoreactors with FZT Y@WDS architecture and the effects of four process factors: the initial concentration of NAP, the initial pH of the solution, the amount of loaded photocatalyst (C_Cat_), and the irradiation time for photocatalytic degradation of NAP under visible light, the Response Surface Method (RSM) was used. The analysis of this activity was performed using the RSM method of design of experiments with Design Expert 11 software. It is believed that this semiconductor nanocatalyst is well suited for photocatalytic activity.

## Experimental method

### Photocatalytic activity of FZT Y@DS smart nanoreactors

FZT Y@WDS smart nanoreactors were used for photocatalytic degradation of NAP. The reactor was fed with 200 mL of a feed containing 100 mg/L NAP for each experiment. In addition, the pH of the reaction medium was adjusted according to the experimental design and the amount of smart nanoreactors with FZT Y@WDS structure added to the reaction medium. The dark experiment was performed for 30 min to reach the adsorption equilibrium concentration, and immediately after the addition of the catalyst, the reactor was protected from light. The irradiation time started with the design of the experiment in the presence of a lamp with visible light (Osram Ultra-Vitalux, 400 W). At the end of each reaction, the sample was removed from solution and centrifuged at 12,000 rpm for 30 min. The final concentration of NAP was measured by HPLC. The amount of degradation of NAP was analyzed using a Thermo Fisher U-HPLC instrument (3,000 Model-Ultimate). All samples were analyzed using the same HPLC method to determine the properties, and the concentrations were confirmed using calibration charts. The removal of NAP (%) was calculated using Eq. 1:

$$NAP\left(\%\right)=\left(\frac{C_{i}-C_{f}}{C_{i}}\right)\times 100$$ (1)

where C_f_ and C_i_ are, respectively, the NAP concentrations after and before the photocatalytic processes.

## Results and discussion

### Structural properties of FZT Y@DS smart nanoreactors

#### Characterization of FZT Y@DS smart nanoreactors

**FIGURE 1:**
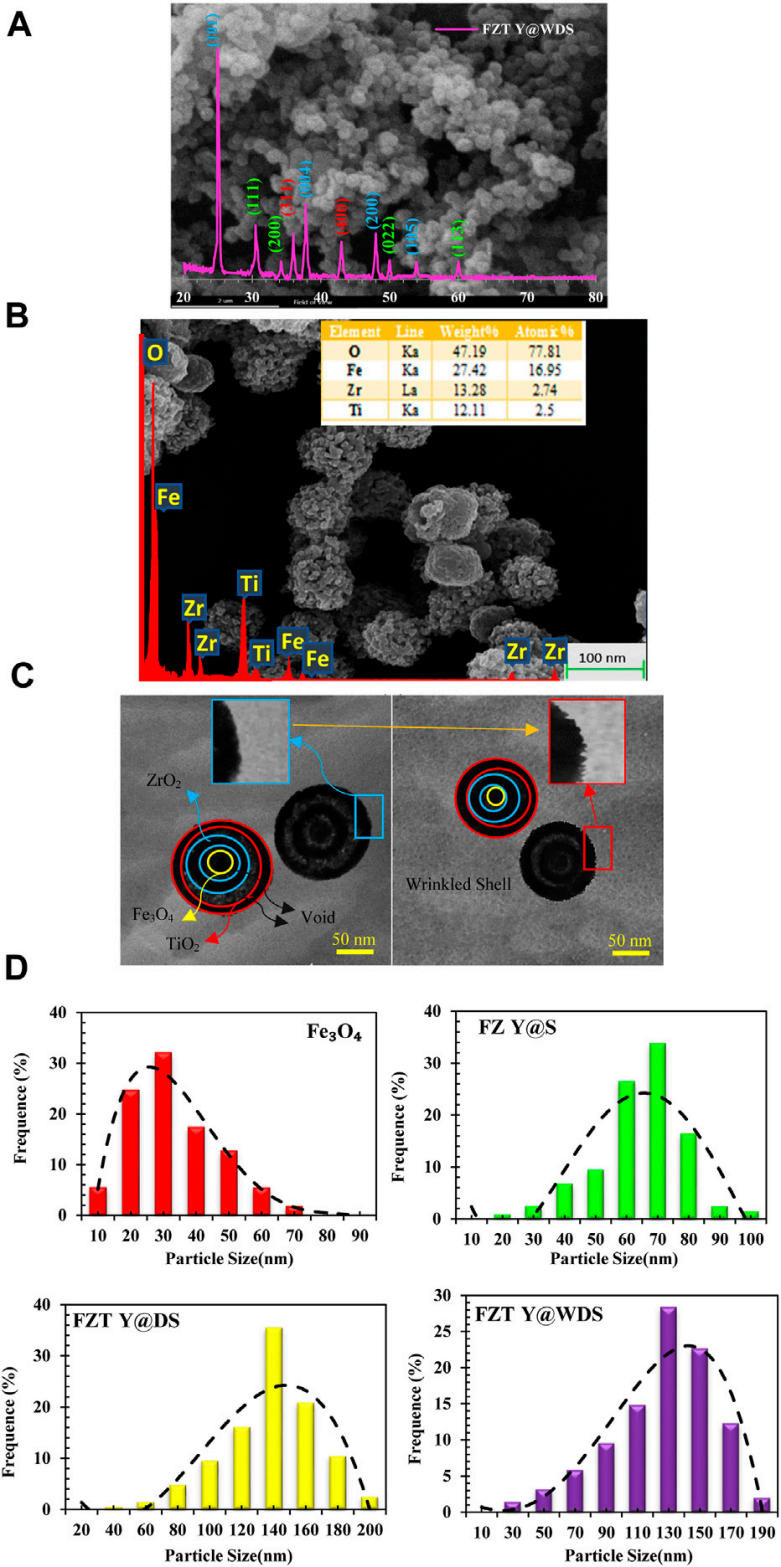
**(A)**. XRD, **(B)**. SEM and EDS analysis **(C)**. TEM images related to structural smart nanoreactors with FZT Y@WDS architecture, **(D)**. the average sizes of Fe_3_O_4_, FZ Y@S, FZT Y@DS and FZT Y@WDS NPs.

To characterize and confirm the structure of the FZT Y@WDS smart nanoreactors, the following analyzes were performed. The X-ray diffraction (XRD) pattern of the smart nanoreactors from FZT Y@WDS was obtained with the Equinox-3000, Inel France (using a Co-Kα X-ray tube with an input voltage of 40 kV and a scanning speed of 1° (1/min) and 2θ intervals (the value is 0.02°)). The crystalline phase information was confirmed based on the JCPDS database. The XRD patterns for the FZT Y@WDS smart nanoreactors sample are shown in Figure 1A.

It was found that FZT Y@WDS NPs form magnetite with well-defined crystallization in a cubic phase, as shown in Figure 1A. Moreover, it is confirmed that the phase structure is anatase with respect to the second TiO_2_ shell, based on the characteristic diffractions of an inverted spinel without impurities for the Fe_3_O_4_ core (Zhuang et al., 2015) and the monoclinic structure for the first ZrO_2_ shell (Heshmatpour and Aghakhanpour, 2011). The Scherrer formula was used to calculate the average crystallite sizes:

$$D=\frac{0.9\times \lambda }{\beta \times \cos\theta }$$ (2)

where θ, diffraction angle; λ is the wavelength of the X-rays; β, FWHM width of the diffraction peak (Klug and Alexander, 1974).

FZT Y@WDS crystals had an average crystallite size of 18.53 nm. In addition to measuring the weight and atomic percentage of the samples, FEI Quanta 200 SEM was used to obtain SEM images, investigate the morphology of the smart structural nanoreactors from FZT Y@WDS, and evaluate the morphology of the smart structural nanoreactors from FZT Y@WDS using EDS or EDAX analysis. Figure 1 shows the results of SEM investigations of nanoparticle size and surface morphology. The microscopic image of the FZT Y@WDS smart structural nanoreactors is shown in Figure 1. According to Figure 1, Fe_3_O_4_ with a spherical structure was synthesized by the solvothermal method, and zirconia with an almost cubic structure as the first shell on the Fe_3_O_4_ core, followed by titanium dioxide with a spherical and almost identical structure, can be detected FZT Y@WDS NPs. The size of the particles is almost the same for each nanoparticle and identical in terms of shape. Y@WDS nanoreactors with smart structure for FZT were analyzed by energy dispersive X-ray spectroscopy (EDS) for the presence of O, Fe, Zr, and Ti. The EDAX spectrum shown in Figure 1B confirms the presence of all these elements. According to the results, Ti: Zr: Fe has an atomic ratio of 2.00: 1.5: 1.00. The synthesized FZT Y@WDS NPs showed characteristic peak lines at 0.52 kV for K_α_ of O and at 0.70, 6.39, and 6.92 kV, which can be assigned to L_α_, K_α_, and K_β_ for Fe, respectively. Furthermore, the EDS data of the synthesized sample indicate the presence of zirconium in the synthesized nanoparticles, confirming its presence in the synthesized NPs (characteristic emission lines of 2.04 and 2.26 kV L_α_ and L_β_). Transmission electron microscopy images (TEM) were acquired using a JEOL 2100 microscope at 120 kV. Samples were prepared by suspending the finely powdered products in 100% pure ethanol under sonication and placing a drop of the suspension on a carbon support coated with a gold grid to allow the ethanol to evaporate naturally before analysis. The yolk@shell structure of the Y@WDS NPs shown in Figure 1C was confirmed.

From the particle size distribution (PSD) diagrams in Figure 1D, the PSD of Fe_3_O_4_, FZ Y@S, FZT Y@DS and FZT Y@WDS NPs are about 30, 60, 140 and 130 nm, respectively, with the average size of FZT Y@WDS smart NPs reduced by about 10 nm due to the wrinkled shell of FZT Y@DS.

**FIGURE 2:**
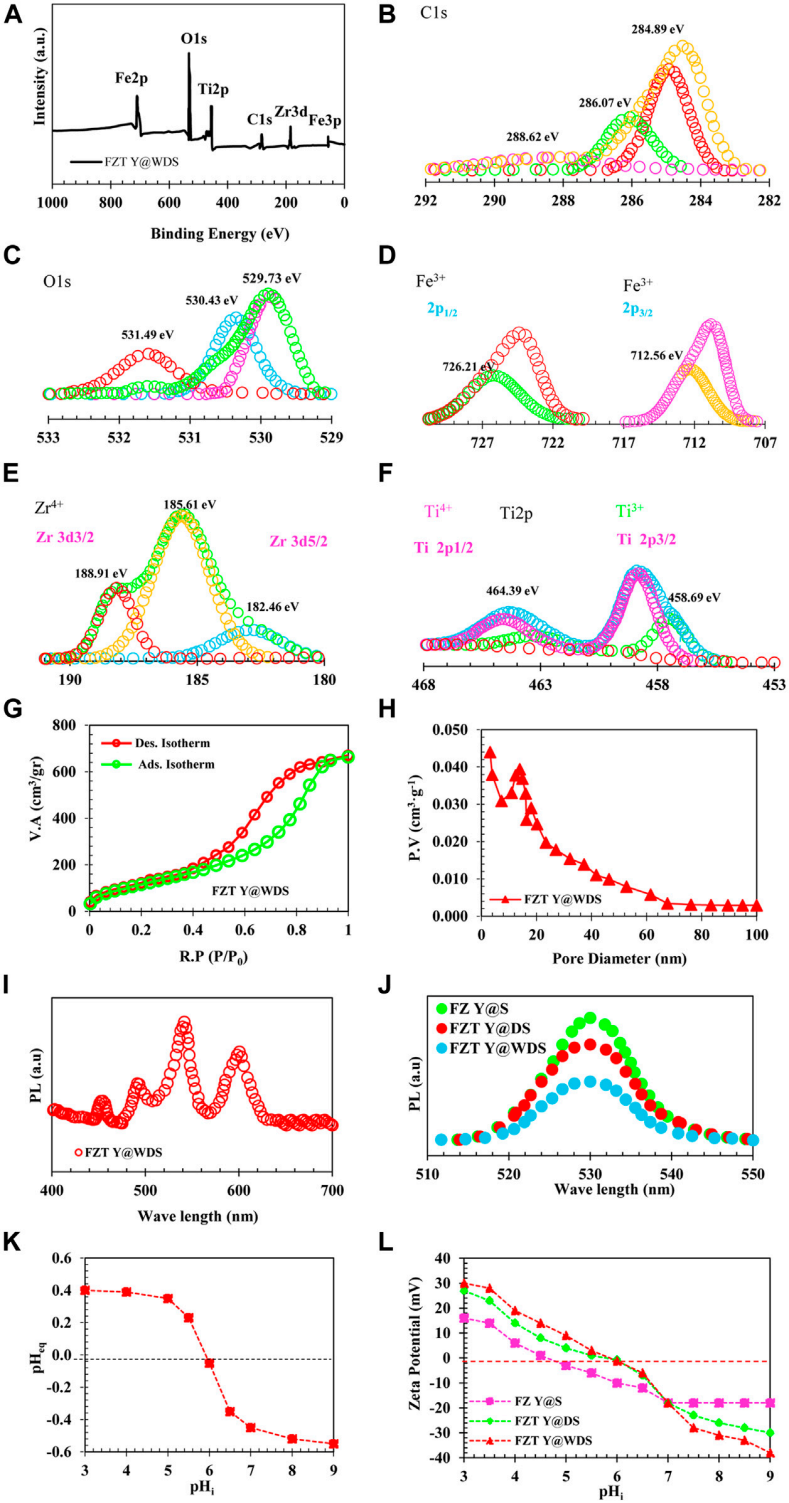
**(A)**. XPS analysis of FZT Y@WDS NPs, **(B)**. C1s, **(C)**. O1s, **(D)**. Fe 2p, **(E)**. Zr 3d, **(F)**. Ti 2p, of level spectrum. of level spectrum. **(G)**. Absorption-Desorption Diagram of FZT Y@WDS NPs, **(H)** Pore Volume of FZT Y@WDS NPs, **(I)**. PL spectra of FZT Y@WDS, **(J)**. PL spectra of samples FZ Y@S, FZT Y@DS and FZT Y@WDS NPs **(K)**. Determination of PZC of FZT Y@WDS NPs **(L)**. The zeta-potential of FZ Y@S, FZT Y@DS and FZT Y@WDS NPs at various pHs.

Surface analyzers (TriStar-II series, Micromeritics Instrument Corporation, United States) were used to determine the texture properties of the Y@WDS NPs. BET (Bruauer-Emmett-Teller) was used to evaluate the specific surface area (SSA) of the Y@WDS NPs. Based on an isothermal absorption and desorption diagram, we calculated the average pore size and pore size distribution using the Barrett-Joyner-Hacienda (BJH) method. The NPs were first degassed at 300 ^°^C for 3 hours before being used in the adsorption-desorption study. The SSA, pore volume, and average pore size of the FZT Y@WDS smart structural nanoreactors were 463 m^2^/g, 0.46 cm^3^/g, and 26 nm, respectively. The N_2_ gas adsorption-desorption isotherms of FZT Y@WDS structural smart nanoreactors are shown in Figure 2G. IUPAC classification IV type adsorption-desorption isotherm can be used to describe this isotherm. The FZT Y@WDS smart nanoreactors from FZT Y@WDS produced residual H3 (Jung and Park, 2000). The synthesis of FZT Y@WDS smart nanoreactors with increased SSA and larger pore volume, consistent with the observations of SEM and TEM, definitely facilitates the achievement of higher photocatalytic activity under solar irradiation. To measure and determine the chemical composition and bond type on the surface of the FZT Y@WDS sample, XPS analysis was performed using a Thermo-Scientific K-Alpha^+^ model from the United States. Figure 2A shows the chemical composition and surface composition of O, Ti, Fe, and Zr in the synthesized FZT Y@WDS NPs. In order to obtain deep fundamental information about the chemical state between the yolk materials (including C and Fe) and the first and second shells of ZrO_2_ and TiO_2_, XPS analysis was carried out to search and find the bonding environment of the FZT Y@WDS smart structural nanoreactors. Figures 2D,E,F show the peaks of the energy links associated with Fe, Zr, and Ti, respectively. Carbon-based pollutants are responsible for the presence of carbon in the FZT Y@WDS NPs. According to Figure 2A, the peaks of O1s, Ti2p, and C1s are at 531.33 eV, 461.08 eV, and 288.35 eV, respectively. According to Figure 2F, Ti 2p1/2 and Ti 2p3/2 have peaks at 464.39 eV and 458.69 eV, respectively. The oxidation state Ti^4+^ is indicated by the peak between Ti 2p1/2 and Ti 2p3/2. As shown in Figure 2E, the surface spectrum of Zr3d contains double peaks for Zr 3d3/2 and Zr 3d5/2 at binding energies of 188.91 eV and 185.61 eV, respectively. The identification of zirconium as a Zr^+4^ oxidation state is indicated by the energy difference of 3.30 eV. A third peak suitable for the shoulder, appearing at the base of Zr 3d3/2, can be attributed to oxygen starvation, which may be due to the misaligned Zr sites of very small ZrO_2_ NPs. The strong exchange between Ti and the elements C and Zr can be attributed to the increased binding energy of Ti2p in the FZT Y@WDS smart structure nanoreactors. Figure 2A shows the spectroscopy of the FZT Y@WDS nanoreactors with smart structure. The electron binding energies of O1, Fe2p, Ti2p, C1s, and Zr3d clearly show the presence of the elements O, Fe, Ti, C, and Zr, proving that FZT Y@WDS nanoreactors with smart structures have been successfully synthesized. According to Figure 2C, the peaks of O1s occur at 531.49 eV and 530.43 eV, corresponding to the bulk oxygen band (Ti-O) at the peak point. In addition to the oxygen present in the surface hydroxyl groups, the stronger peak at 531.49 eV is due to the carbonate species. O1 exhibits the same reduction in binding energy as TiO_2_ (Figure 2C), again indicating oxygen atomic vacancies in the O1s XPS spectrum. Figure 2B shows the C1s spectrum of the FZT Y@WDS nanoreactors with smart structure found in three charged peaks at 284.89, 286.07, and 288.62 eV. FZT Y@WDS smart structure nanoreactors are used as photon sensitizers to increase the absorption of visible light by residual carbon, causing the peak at 284.89 eV. FZT Y@WDS nanoreactors exhibit two peaks at 288.62 eV and 284.89 eV, which are likely due to adsorption of oxygen-bonded species on the surface. Therefore, the smart structural nanoreactors from FZT Y@WDS harbour multiple carbon species on their surface.

The composition of the photocatalyst can be determined by XPS analysis, but the deviation of the band gap of the heterojunction can also be determined by XPS analysis. VB can be accurately determined by measuring the deviation of the core surfaces before and after contact. The deviation (displacement) of VB is calculated according to Eq. 3 or Eq. 4, so that CB is calculated according to Eq. 5. The exact location of the bandgap structure relative to the NHE is useful for investigation and use in appropriate applications, as mentioned earlier.

$${∆E}_{V}=\left(E_{VBM}^{A}+E_{CL}^{A/AB}-E_{CL}^{A}\right)-\left(E_{VBM}^{B}+E_{CL}^{B/AB}-E_{CL}^{B}\right)$$ (3)

$${∆E}_{V}=\left(E_{CL}^{A/AB}-E_{CL}^{B/AB}\right)-\left[\left(E_{CL}^{A}-E_{VBM}^{A}\right)-\left(E_{CL}^{B}-E_{VBM}^{B}\right)\right]$$ (4)

$${∆E}_{C}=E_{g}^{A}+{∆E}_{V}-E_{g}^{B}$$ (5)

Where ΔE_V_ is the deflection (displacement) of VB (eV) and ΔE_C_ is the deflection (displacement) of CB (eV). Figures 2A,B shows the results of XPS analysis and bandgap alignment of FZT Y@WDS. The VB edges of FZ Y@S, FZT Y@DS, and FZT Y@WDS are 2.9, 2.85, and 2.80 eV, respectively, as shown in Figures 2C,D. When Fe_3_O_4_, ZrO_2_, and TiO_2_ are combined, changes (shifts) in Ti 2p and Zd 3d core levels occur. When only the DRS and Mott-Schottky analysis is determined and performed, these shifts in the core levels cannot be detected (Figure 2E). These changes (shifts) in the core levels can be used to determine the deflection VB and the deflection CB (Eqs. 4–6) (Figure 2F). A comparison of VB and CB before and after contact shows that VB and CB decrease when Fe_3_O_4_, ZrO_2_, and TiO_2_ come into contact. In this case, electrons transferred from ZrO_2_ to TiO_2_ (band structure increases) or Fe_3_O_4_ (band structure decreases) could explain the results. The binding energies (531.33 eV, 530.43 eV, and 529.73 eV) are observed with respect to O1s and assigned to the O^2-^ lattice, the OH^−^ lattice, and adsorbed H_2_O molecules, similar to previous studies. This suggests that there is a layer of TiO_2-x_ (OH)_2x_ and ZrO_2-x_ (OH)_2x_ on the surface of the TiO_2_ and ZrO_2_ shells.

Determination of the photoluminescence (PL) property of the samples in terms of emission intensity in different wavelengths after their excitation with photons of a certain wavelength was performed by PL analysis or PL spectroscopy (model G9800A from Agilent). PL spectra were measured using a Cary Eclipse fluorescence spectrometer (Agilent Technologies) (at room temperature, the wavelength range of 400–800 nm at an excitation wavelength of 293 nm). Figure 2I shows the analysis of the PL spectrum to calculate the efficiency of charge separation at the sample interface. Figure 2I shows the PL spectrum of FZT Y@WDS. The activation of photocatalysts is strongly influenced by their light absorption. Moreover, photocatalytic activity is closely related to the spectrum and intensity of PL. The highest intensity of all the samples can be seen in Figure 2J. As a result of the yolk@shell structure, increased photon harvesting area, decreased nanoparticle energy band gap, oxygen vacancies, defects in surface states, and other structural impurities in the samples FZ Y@S, FZT Y@DS, and FZT Y@WDS, a peak can be observed in the visible region. The high PL intensity of FZ Y@S clearly indicates that the surface states of ZrO_2_ are much lower, and therefore the simple transfer of electrons from VB to CB of ZrO_2_ can occur even with a low energy laser excitation source (Ramakrishna et al., 2004). The synergistic effect of FZT Y@WDS nanoreactors with smart structure and the possibility of more (-Fe-O-Zr-), (-Ti-O-Fe-), and (-Ti-O-Zr-) by the PL spectrum of pure catalysts Fe_3_O_4_ in the core and ZrO_2_ in the first shell and TiO_2_ in the second shell are physically confirmed (Wu et al., 1984). The results of Figure 2J PL show the peaks of Fe_3_O_4_ photocatalysts, FZ Y@S, FZT Y@DS and FZT Y@WDS at 543 nm with the above crystallinity. The fluorescence intensity of the Fe_3_O_4_ composite photocatalyst decreases after the addition of the first ZrO_2_ shell. The separation of photoexcited electrons and holes may be enhanced by the ZrO_2_ shell’s electronic properties. In addition, ZrO_2_ can maintain absorption in the visible range due to its yolk@shell structure and its ability to trap photons. Compared to the smart structural nanoreactors FZT Y@DS, the nanoreactors FZT Y@WDS have significantly improved PL properties. At the excitation wavelength of 360 nm, the ZrO_2_ electrons cannot jump from the VB to the CB, so the PL emission of ZrO_2_ NPs does not increase upon excitation. However, by reducing the energy band gap with the yolk@shell structure and the presence of voids between the shell and the core of this structure, the problem could be solved. This theory is roughly supported by the waveform shown in Figure 2J. Moreover, coating TiO_2_ on FZ Y@S as a second shell significantly increases the surface area and photon emission and harvesting in FZT Y@WDS smart structure nanoreactors, suggesting that TiO_2_ as a second shell layer can increase luminescence. While ZrO_2_ and TiO_2_ alone and due to their chemical inertness prevent the efficient separation of photoexcited carriers. Figure 2K describes the optimal pH (=5.8) for nanoreactors with the FZT Y@WDS smart structure as a function of the type of contaminant and PZC. Catalyst surfaces are neutral at pH = pH_PZC_, while negatively and positively charged surfaces are considered at pH>pH_PZC_ and pH < pH_PZC_, respectively. Both adsorption rate and photocatalytic reaction rate are affected by pH, which is obvious. Zeta potential measurements confirmed that stable colloids of Fe_3_O_4_ NPs, FZ Y@S and FZT Y@WDS were successfully obtained, and Figure 2L shows the zeta potential behavior of these suspensions as a function of pH. Plotting the zeta potential *versus* pH in Figure 2L shows that the isoelectric points for Fe_3_O_4_, FZ Y@S and FZT Y@WDS are at pH 4.6, 5.6 and 5.8, respectively. Figure 2L also shows that the surface zeta potential of the FZT Y@WDS smart structural nanoreactors is higher than that of FZ Y@S and Fe_3_O_4_. The mesoporous coating generates a continuous positive charge in the lower pH range (below the isoelectric point), which could be due to the presence of protonated surface hydroxyl groups (Ti-OH^2+^) and (Zr-OH^2+^). Therefore, as the positive charge density on the adsorbent surface increases at low pH, the zeta potential value increases, while at higher pH, the proton surface sites decrease and the zeta potential becomes more negative. It should be noted that the zeta potential of the particles is positive at low pH, indicating the absorption of H^+^ ions, which can change the surface free energy of ZrO_2_ and TiO_2_ and create a thermodynamic barrier between anatase and rutile phases. It also reduces the tetragonal and monoclinic phases, allowing martensitic transformation. The high value of the zeta potential for the FZT Y@WDS smart structural nanoreactors in acidic environment indicates the high stability of the NPs in suspension with an initial zeta potential of +30 mV and an isoelectric point (IEP) at pH = 5.8. It is known that electrostatic stabilization is the most common way to stabilize aqueous colloidal suspensions and that the zeta potential of the particle surface is affected by changes on the nanoparticle surface, mainly due to the fact that the oxide surface can be strongly oxidized by hydrogen or hydroxyl ions from the solution, as shown by the reversal of the zeta potential between pH 5.5 and 6.5. However, the interaction between two particles depends on the balance between electrical repulsion and Van der Waals attraction. In aqueous solution, the surface charge of the intelligently structured nanoreactors FZT Y@WDS varies as a function of pH.

#### The effect of FZT Y@WDS smart NPs on electrochemical properties

Electrochemical analyzes, including cyclic voltammetry and electrochemical impedance spectroscopy, were performed to investigate the effects of the different structures of the samples on performance and electrochemical properties, including charge transfer.

#### Electrochemical properties of FZT Y@WDS smart NPs

**FIGURE 3:**
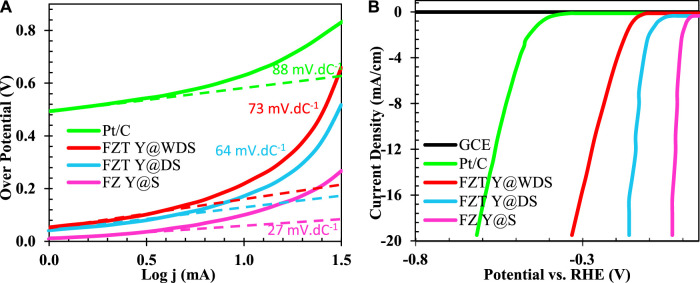
Diagram of **(A)**. Polarization and **(B)**. Tafel related to FZ Y@S, FZT Y@DS and FZT Y@WDS samples compared to Pt/C electrode.

To gain a better understanding of the irregular surface layer of the FZT Y@WDS smart structural nanoreactors, the surface reactivity was investigated by studying the electrocatalytic activity during the production of hydroxyl ions. Figure 3A shows the polarization curves in which the normalized (standard) current density is plotted against the applied potential without iR correction. The Y@WDS FZT sample has a much lower overvoltage (ƞ) (−149.6 mV) at a current density of 15 mA.cm^-2^ (vs RHE, as below) than sample Y@DS FZT (−576.6 mV). The corresponding Tafel slope of the FZT Y@WDS sample is 64 mV.dec^−1^ (Figure 3B), which is lower than that of the FZT Y@DS sample (88 mV.dec^−1^), indicating higher activity toward hydroxyl ion production. These results indicate that the disordered layer on the surface of FZT Y@WDS can significantly enhance the surface response to hydroxyl ion generation. It is hypothesised that increasing the number of electron carrier trapping sites (i.e., oxygen vacancies) enhances electron transfer, which effectively lowers the energy barrier for proton reduction and activates surface reactivity for hydroxyl ion formation.

#### Cyclic voltammetry test

**FIGURE 4:**
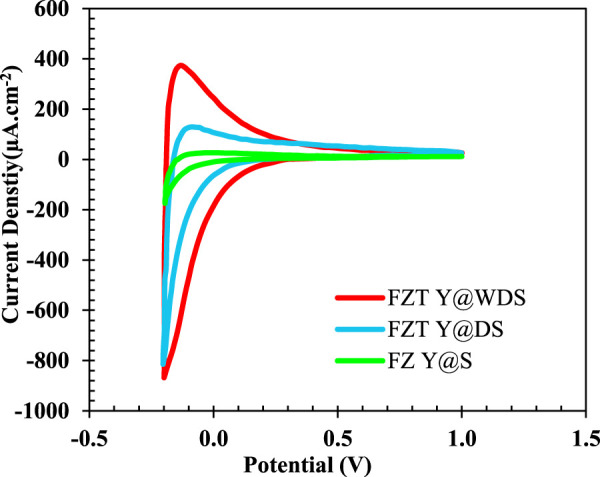
Cyclic voltammetry curves of FZ Y@S, FZT Y@DS and FZT Y@WDS samples (with a sweep rate of 0.1 V/s).

The electrochemical properties of Fe_3_O_4_, FZ Y@S, FZT Y@DS, and FZT Y@WDS samples with a surface area of 1 cm^2^ in contact with 0.5 M sodium sulfate electrolyte were studied by cyclic voltammetry; the resulting plots are shown in Figure 4. As can be seen from Figure 4, structurally coated samples have higher current density than the sample with Fe_3_O_4_ NPs. The coating in the structure of the nanoreactors and the formation of a heterojunction form a new impurity level above the valence band of the samples, which helps to transfer more electrons, i.e., increase the transfer rate of electrons from the valence band to the conduction band and decrease the recombination rate and electron loss. gives, resulting in more current. With the addition of the coated layer in the designed yolk@shell structure, the rate of charge separation and electron transfer increases and the recombination decreases, which increases the current density.

#### Electrochemical impedance spectroscopy

**FIGURE 5:**
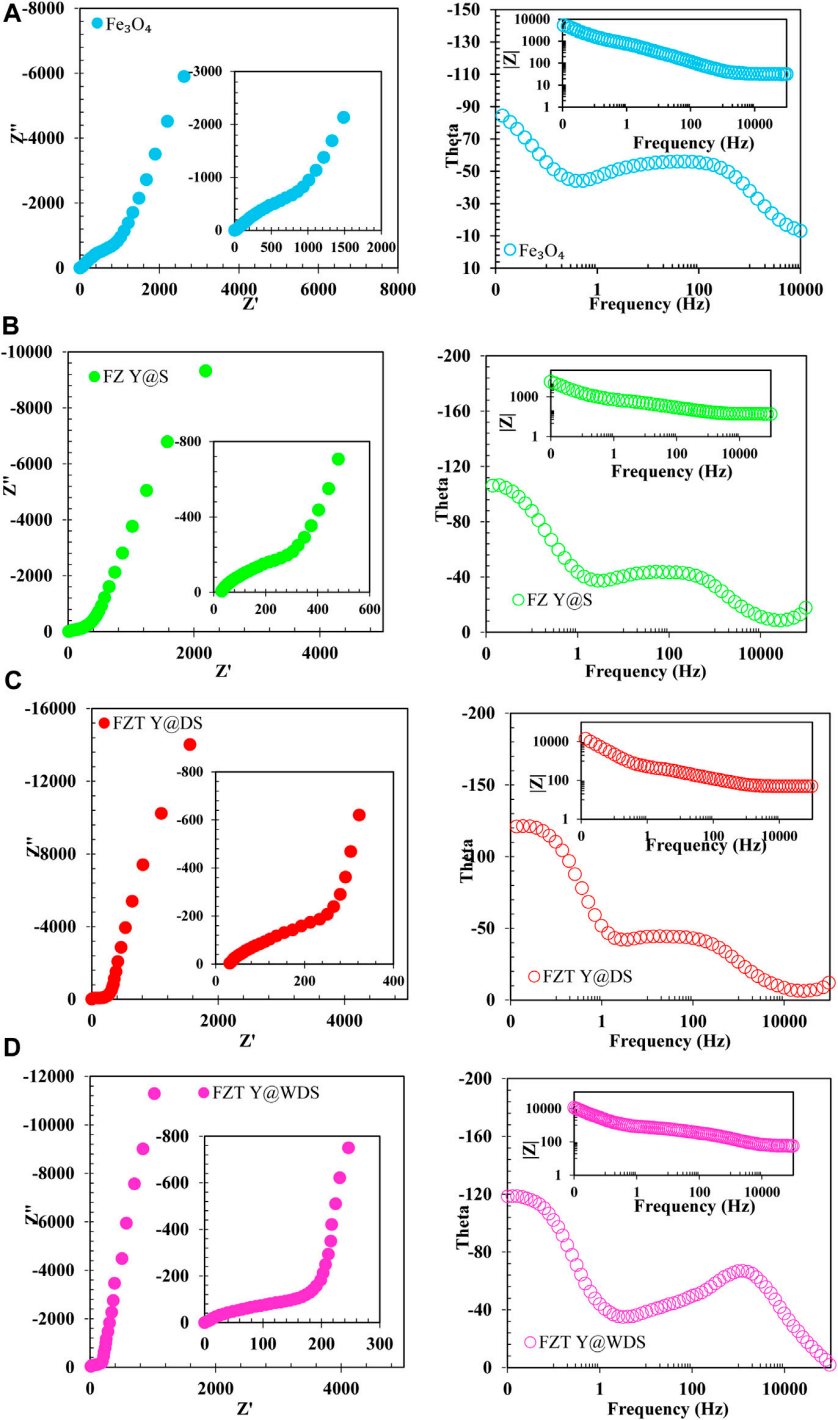
**(A)**. Nyquist, Bode and phase diagrams of **(A)**. Fe_3_O_4_, **(B)**. FZ Y@S, **(C)**. FZT Y@DS and **(D)**. FZT Y@WDS

Electrochemical impedance spectroscopy (EIS) analyzes were performed to integrate the enhanced energy storage properties of the yolk@shell sample. Figure 5 shows the Nyquist diagram of yolk@shell for high (intrinsic) frequencies from the EIS experiments. In the high frequency range, there is a semicircular arc, in the low frequency range a straight line. At low frequencies, the slope of the line is similar, indicating low diffusion resistance of the ions, which is close to the behavior of an ideal capacitor. The ESR can be evaluated as intersecting the real axis of the Nyquist diagram. The ESR of yolk@shell (∼1.2 Ω) is lower than that of other samples (∼1.7 Ω). In the high frequency region, the diameter of the semicircle can be used to measure the charge transfer resistance (R_ct_). A comparison of FZT Y@WDS and other samples shows that FZT Y@WDS has a lower R_ct_ (∼0.1 Ω) than other samples (∼5.4 Ω). The equivalent series resistance (ESR) is connected in parallel with a constant phase element (CPE_1_) in parallel with the charge transfer resistance (Rct_1_). A constant phase element (CPE_2_) is connected in series in parallel with the charge transfer resistance (R_ct2_) and a constant phase element (CPE_3_) is connected in series in parallel with the charge transfer resistance (R_ct3_). To further investigate the resistance of the individual parts of the FZT Y@WDS electrode system of the yolk@shell hollow nanospace structure, the EIS of the yolk@shell hollow nanospace structure of the FZT-Y@WDS electrode was modeled by the modified Randles equivalent circuit (Figure 5C). To investigate the effects of yolk@shell structural architecture on the interfacial properties of FZT Y@WDS electrodes with a surface area of 1 cm^2^, electrochemical impedance spectroscopy analysis was performed in 0.5 M sodium sulfate solution. The data obtained from the experiment were fitted with the mentioned equivalent circuit, and the values of the parameters of the equivalent circuit are listed in Table 1. The mixed level impedance plots (Nyquist, Bode and phase) obtained from the experiment and the fitted plots are shown in Figure 5C,D. Figures 5A,B belong to sample FZT Y@DS and Figures 5C,D also belong to sample FZT Y@WDS (with the same synthesis parameters). The Nyquist diagram of the samples consists of two parts: Charge transfer and mass transfer (diffusion). The curve of the charge transfer part is a more compact semicircle than the perfect semicircle. For this reason, we used a constant phase element instead of an ideal condensor element to model the surfaces of the porous walls, which are heterogeneous and non-ideal. The shape of the Nyquist diagram of the two samples is the same, and the only difference is in the size and magnitude. Comparing the semicircles of the two samples FZT Y@WDS and FZT Y@DS, we find that the semicircle of sample FZT Y@WDS has a smaller diameter. Since the diameter of the semicircle is a measure of the charge transfer resistance, the FZT Y@WDS sample has a lower charge transfer resistance. In other words, the rate of charge transfer and conduction increases and the rate of recombination decreases. Table 1 shows that the FZT Y@WDS sample has a higher CPE-T value than the FZT Y@DS sample (about three times). This shows that the FZT Y@WDS sample has a higher charge conductivity. The resistivity of the FZT Y@DS sample is 1,054 Ω and that of the FZT Y@WDS sample is 100 Ω. Therefore, the charge transfer resistance of FZT Y@DS is greatly reduced due to the wrinkled shell architecture. From the Nyquist, Bode and phase diagrams of the mass transfer (diffusion) section and the data in Table 1, it can be seen that the W-R value of the FZT Y@WDS sample is lower than the W-R value of the FZT Y@DS sample, so it has a lower diffusion resistance. Now we investigate the effects of the nanoreactor structure on the electrochemical properties and the interface of the nanoreactors with EIS. Again, the graphs obtained from the experiment were fitted using the aforementioned equivalent circuit, and the values of the parameters of the equivalent circuit are listed in Table 1; Figures 5C,D show the Nyquist and Bode and phase diagrams, respectively, of sample FZT Y@DS. A comparison of the semicircles of all three samples FZT Y@DS, FZT Y@WDS and FZ Y@S shows that sample FZT Y@WDS has a smaller diameter and thus a lower charge transfer resistance than the other samples. According to Table 1, the charge transfer resistance of FZT Y@DS, FZT Y@WDS and FZ Y@S samples are 147, 100 and 204 $\Omega /{cm}^{2}$ , respectively. The FZT Y@WDS sample has higher CPE-T value and lower W-R value than the other samples, which confirms the high conductivity and low penetration resistance of FZT Y@WDS sample.

**TABLE 1:** EIS data fitting results in open circuit conditions for Fe_3_O_4_, FZ Y@S, FZT Y@DS and FZT Y@WDS samples.

Sample	$R_{s}/\Omega .{cm}^{2}$	$CPE-T/\mu .F.{cm}^{-2}$	$CPE-P/\mu .F.{cm}^{-2}$	$R_{ct}/\Omega .{cm}^{2}$	$W-R/\Omega .{cm}^{2}$	$W-T/\Omega .{cm}^{2}$	W-P
F	13.767	6.83725E-05	0.7012	714	1041	0.9813	0.300435
FZ Y@S	17.9835	0.000107755	0.68172	230.8	569.2	0.352615	0.325955
FZT Y@DS	17.7345	0.000104665	0.73322	182.9	476.55	0.26217	0.34613
FZT Y@WDS	6.3879	0.00020191	0.80736	100.6	363.1	0.69765	0.43259

Adding the second shell and folding the shell increases the void, surface area, and available anatase TiO_2_ phase, decreasing the charge transfer resistance. However, if the calcination time is increased, the length of the nanoreactors exceeds the optimal limit and the charge conductivity decreases, i.e., the charge transfer and recombination resistance increases. Therefore, long nanoreactors are not suitable for charge transfer because they slow down the charge transfer.

It can be concluded that the addition of the second shell and the wrinkling of the shell means an increase in the free trapping space, and the size of the surface area is one of the important and influential parameters for the electrochemical properties of FZT Y@WDS electrodes.

Therefore, an optimal length should be selected to transfer the maximum charge. EIS analysis confirms the results of CV analysis.

### Investigating electrochemical impedance spectroscopy

**FIGURE 6:**
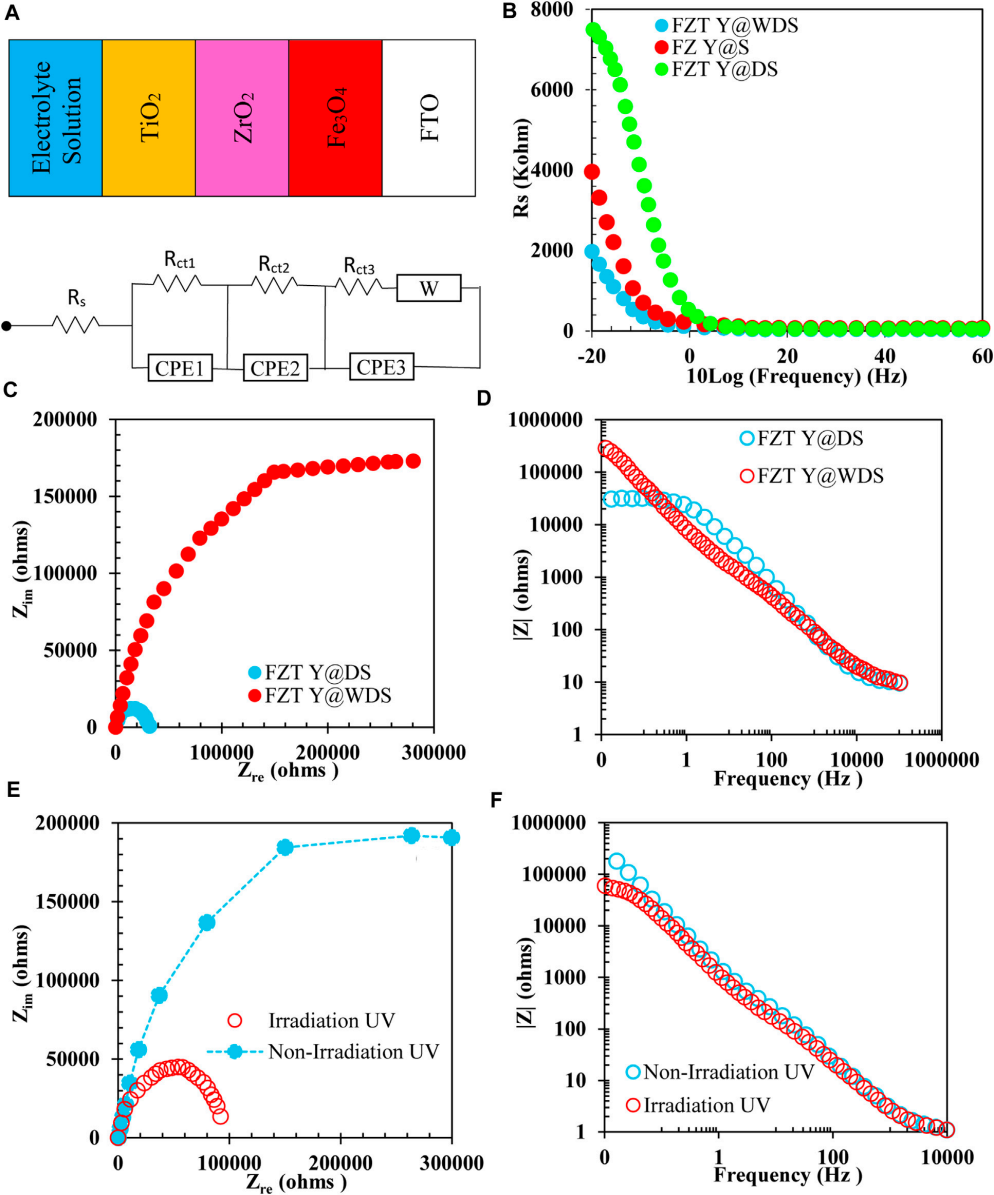
**(A)**. Equivalent circuit of FZT Y@WDS sample, **(B)**. Resistance curve in terms of 10log(frequency) of FZ Y@S, FZT Y@DS and FZT Y@WDS samples, **(C)**, **(D)**. Electrochemical impedance spectroscopy Nyquist and Bode corresponding two samples of FZT Y@DS and FZT Y@WDS, **(E)**, **(F)**. Electrochemical impedance spectroscopy of FZT Y@WDS nanoreactors in the presence and absence of visible light.

For further investigation, the equivalent circuit of the FZT Y@WDS sample was drawn using ZView software and is shown in Figure 6A along with a schematic of the sample. Electrochemical impedance spectroscopy data are also shown in Table 2; Figure 6B shows the resistivity curve in terms of 10log frequency of Fe_3_O_4_, FZ Y@S, FZT Y@DS and FZT Y@WDS samples, as can be seen, by adding the first and second shells in the yolk@shell structure, the electrical conductivity has increased. The reason is the presence of oxygen structural defects on the surface of nanoreactors and the presence of trivalent titanium and zirconium ions on the shells, and on the other hand the presence of oxygen-containing functional groups such as hydroxyls, which have a covalent bond to the surface, causing the creation of electrons useful and as a result of the reduction of electrical conductivity, these functional groups play the role of insulation. Therefore, the reduction of functional groups can help to increase the electrical conductivity. As shown in Figures 6C,D, electrochemical impedance spectroscopy of FZT Y@DS and FZT Y@WDS samples with the first ZrO_2_ shell in the FZ Y@S nanoreactor and the second TiO_2_ shell in the FZT Y@DS nanoreactor and the wrinkling of the FZT Y@WDS nanoreactor-Shell show a positive role in reducing the resistance of NPs that one of the reasons for reducing the resistance can be considered the positive role of the structural design in reducing the energy band gap and preventing the recombination of electrons and holes in these nanoreactors. Figures 6E,F shows the comparison of electrochemical impedance spectroscopy of Nyquist curves and resistance with respect to frequency of two samples of FZT Y@DS and FZT Y@WDS. As can be seen, the sample FZT Y@DS has more resistance than the sample FZT Y@WDS. Considering the stability of the chemical compounds used for the structure of these two nanoreactors, this increased resistance is due to the conditions of the synthesis process and the optimal design of the yolk@wrinkled shell architecture. To determine the cause of this increase in resistance, the mechanism of bandgap energy is investigated. Library studies of the capacitance band VB and conduction band CB of the three materials investigated in this study have shown that Rs is related to the resistance of the solution and that the magnitude of R_s_ is nearly constant in all experiments due to the stability of the solution.

**TABLE 2:** Data related to electrochemical impedance spectroscopy of two samples FZT Y@DS and FZT Y@WDS

Sample	R_s_(Ω)	CPE_1_(F)	R_1_(Ω)	CPE_2_(F)	R_2_(Ω)	CPE_3_(F)	R_3_(Ω)
FZT Y@DS	9.81	1.11 × 10^−5^	5.73×10^5^	1.40 × 10^−5^	2,587	9.40 × 10^−5^	1,543
FZT Y@WDS	9.59	4.63 × 10^−6^	30,887	4.93 × 10^−6^	14,560	1.93 × 10^−5^	3,498

As can be seen from the equivalent circuit of the sample and the data in the table, both samples have CPE capacitance resistance and charge transfer resistances R_1_, R_2_ and R_3_, which are in order to adapt the proposed graph of the zview software related to the Fe_3_O_4_ core and shell are ZrO_2_ and TiO_2_. Adding the second layer, i.e., the TiO_2_ NPs, and wrinkling the shell structure with the sample data added a CPE capacitor resistor and another R_3_ charge transfer resistor to the circuit. As can be seen, not only the charge transfer resistance and the new capacitor CPE_3_, R_3_ decreased, but also the amount of CPE_2_, R_2_ and CPE_1_, R_1_, and in general, the total resistance of the circuit decreased from $76.5\times 10^{4}$ Ω to $2.8\times 10^{4}$ Ω.

Figure 6 shows a schematic diagram of the comparison of these energy levels. According to this mechanism, when the ZrO_2_ shell is placed in the nanoreactor with FZ Y@S structure, the electron can be easily transferred from the conduction band of ZrO_2_ to Fe_3_O_4_ due to the higher energy level of ZrO_2_ compared to Fe_3_O_4_, but when TiO_2_ is placed as the second shell, the electron transfer is unhindered due to the lower energy level of TiO_2_ compared to ZrO_2_ and does not act as an insulating barrier and reduces the resistance. To overcome the energy barrier, the electron must be supplied with energy. This energy can be in the form of heat or electromagnetic waves. Electromagnetic waves also have energy. The energy of electromagnetic waves can be calculated according to their wavelength using Eq. 6 (Planck’s relation).

E=h.c/λ (6)

where E is the wave energy (j), (h) is a constant number called Planck’s constant, c is the speed of light (m/s) and λ is the wavelength of light (m). As can be seen in this relationship, the energy of each wave has an inverse relationship with its wavelength. According to what has been said so far, by generating energy in a semiconductor by visible light, it is possible to excite and move electrons and thus generate current and reduce resistance. According to the above research, the reason for the high resistance of the FZT Y@DS sample compared to the FZT Y@WDS sample is related to the generated potential barrier. On the other hand, the charge transfer resistance in the FZT Y@WDS architecture was decreased due to the wrinkling in the shell. To prove this, this sample was irradiated with visible light. Figure 6 shows a comparison of the electrochemical impedance spectroscopy of FZT Y@WDS nanoreactors in the presence and absence of light. As can be seen, irradiation with visible light greatly reduced the resistance of the sample, which may be evidence that electrons overcome the resistance barrier in the presence of visible light as an external factor.

In the current study, FZT Y@WDS nanoreactors were synthesized and then irradiated with visible light, and the electrical properties of ZrO_2_ and TiO_2_ were compared. The energy band gap studies of the synthesized FZ Y@S NPs revealed that it has a lower band gap than ZrO_2_. Therefore, the effect of adding ZrO_2_ to Fe_3_O_4_ on the architectural structure of yolk@shell was investigated, and the results showed that the addition of ZrO_2_ shell decreased the energy band gap and also increased the electrical conductivity. Finally, the effect of adding TiO_2_ shells to FZ Y@S nanoreactors was investigated. The addition of TiO_2_ also decreased the band gap of this FZT Y@DS nanoreactor, but according to the equivalent circuits of these two nanocomposites, the addition of TiO_2_ NPs to In FZ Y@S sample added a capacitive resistance and a charge transfer resistance to the circuit, which increased the total resistance and consequently decreased the electrical conductivity of the circuit. Investigation of the mechanism of this phenomenon led to the conclusion that the reason for the high resistance of the FZT Y@DS sample compared to FZ Y@S is related to the potential barrier created due to the higher energy level of ZrO_2_ compared to TiO_2_, which gives off energy Visible light can overcome this potential barrier and increase the electrical conductivity.

#### UPS analysis

Electron transfer in heterojunctions is predicted based on the Fermi energy level (E_F_), which is an important part of the band structure. VB and E_F_ of photocatalysts are recorded using UPS. In UPS the horizontal axis intersects the spectral tangent and the intersection is the relative position of VB, when E_F_ is zero electron volts (0 eV). This approach allows us to visualize the VB, CB, E_F_, and trapping modes of a photocatalyst compared to NHE by combining these techniques. In addition, Eq. 7 allows the direct determination of the work function (φ). The work function (φ) (Eq. 8) is equal to the difference between E_vacuum_ (vacuum energy) (at 4.5 V vs NHE) and the energy function (E_F_). To verify that the results are consistent, UPS can be used. The electron transfer occurs between photocatalysts with different work functions (from lower work function to higher work function) when photocatalysts are combined to form heterojunction photocatalysts. Heterojunction photocatalysts irradiated with light can induce internal electric fields that support or prevent further electron transfer. For this reason, there are photocatalysts with S-scheme structures.

φ=hv−∆E (7)

$$\varphi =E_{vacuum}-E_{F}$$ (8)

**FIGURE 7:**
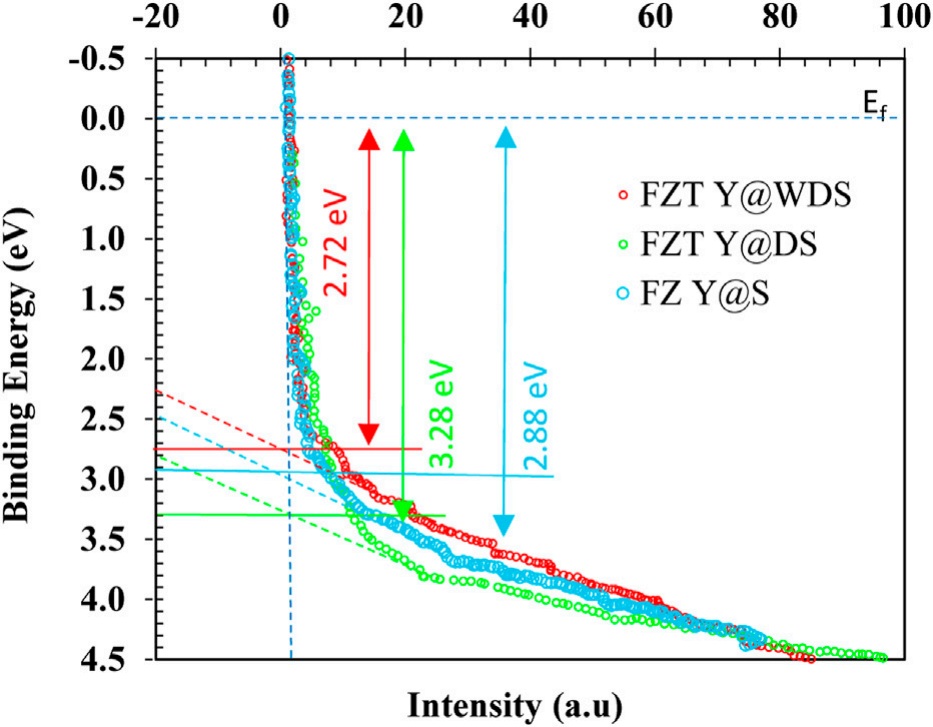
UPS of FZT Y@S, FZT Y@DS and FZT Y@WDS.

UPS the results for Fe_3_O_4_, ZrO_2_ and TiO_2_ as well as FZT Y@WDS in Figure 7 show the position of the highest occupied molecular orbital and VB in relation to E_F_. By combining the results of DRS and Mott-Schottky analysis, the working power of these materials can be calculated by the difference between the vacuum level and E_F_. To compensate E_F_, electrons are transferred from a material with a lower work function (ZrO_2_) to a material with a higher work function (TiO_2_) and (Fe_3_O_4_). This corresponds to the work function of FZT Y@WDS in Figure 7, and the band alignment of the material before and after contact is shown in Figure 7. The transfer of electrons from ZrO_2_ to TiO_2_ and Fe_3_O_4_ bends the band and forms an electric field at the interface of FZT Y@WDS. This is a direct method to validate the latest type of photocatalysts known as S-scheme.

#### Photoelectrochemical (PEC) measurement

**FIGURE 8:**
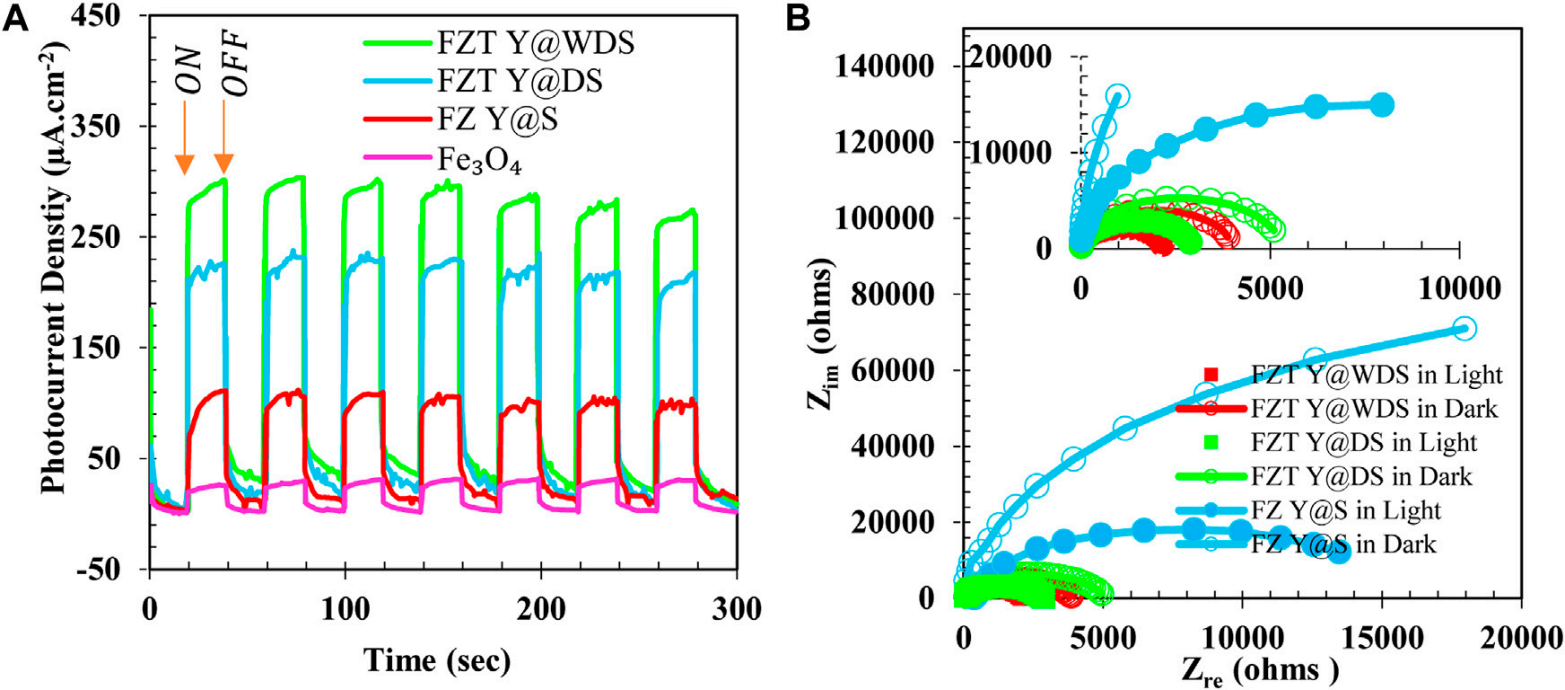
Transient photocurrent response Fe_3_O_4_, FZ Y@S, FZT Y@DS and FZT Y@WDS **(A)** and EIS Nyquist plots **(B)** of FZ Y@S, FZT Y@DS and FZT Y@WDS in light and dark.

To further investigate the features and characteristics of the separation and transport of light-generated electric charge, transient photocurrent measurements were performed under simulated sunlight and darkness with an on/off pattern for 20 s, as shown in Figure 8A. Pure Fe_3_O_4_, pure ZrO_2_, and pure TiO_2_ samples generated current densities of 20 μA.cm^-2^, 30 μA.cm^-2^, and 25 μA.cm^-2^, respectively, under light, while no current was generated in the dark, indicating that the activation of Fe_3_O_4_, TiO_2_, and ZrO_2_ by light is confirmed. By forming a heterojunction of pure Fe_3_O_4_, pure ZrO_2_ and pure TiO_2_, i.e., FZT Y@DS, the generated luminous current density is 65 μA.cm^-2^, which is more than that of pure Fe_3_O_4_, ZrO_2_ and TiO_2_. The generated light current density increased to more than 130 μA.cm^-2^ due to the wrinkling of the shell in the Y@ WDS FZT structure. This increase in current density is mainly attributed to the efficient generation of electrons (e^−^) and holes (h^+^) by efficient light absorption in FZT Y@WDS, which is confirmed by the results of UV-vis DRS analysis and the efficient electric charge separation due to the formation of heterogeneous bonds. (Heterojunction) between Fe_3_O_4_, ZrO_2_ and TiO_2_ is demonstrated. In addition, the presence of voids between the Fe_3_O_4_ core and the wrinkled shells of ZrO_2_ and TiO_2_ also contributes to efficient charge separation due to high conductivity and helps in the movement of charges from one semiconductor to another. Such efficient electrical charge separation can lead to higher photocatalytic efficiency. Electrochemical impedance spectroscopy (EIS) is used to further investigate the mechanism of charge transfer at the surface of the synthesized samples. Nyquist EIS curves of FZ Y@S, FZT Y@DS, and FZT Y@WDS were measured in dark and light environments; the results are shown in Figure 8B. The radius of the EIS semicircles corresponds to the total resistance of the electric charge transfer. All samples show lower charge transfer resistance in simulated light than in the dark. FZ Y@S exhibits high charge transfer resistance in light controlled by the semicircle radius, resulting in lower charge separation efficiency. By forming a heterogeneous compound (heterojunction) of Fe_3_O_4_, ZrO_2_ and TiO_2_, the charge transfer resistance of FZT Y@DS and FZT Y@WDS is greatly reduced in light, indicating efficient charge transfer by heterojunction. Moreover, the incorporation of pure TiO_2_, pure Fe_3_O_4_ and pure ZrO_2_ and the void space between the Fe_3_O_4_ core and the ZrO_2_ and TiO_2_ shells, which forms the wrinkled shell FZT Y@WDS, further reduces the charge transfer resistance in light, as shown by the semicircle radius in Figure 8B. From this, it can be seen that the FZT Y@WDS structure operates with a wrinkled shell with a semicircular radius, as shown in Figure 8B. This shows that the empty space in the wrinkled yolk structure of a conductor helps to separate and transfer the photogenerated electric charge (e^−^ and h^+^) from both semiconductors.

By incorporating ZrO_2_, TiO_2_, and Fe_3_O_4_ to form FZT Y@WDS, an increase in surface area is observed compared to FZT Y@WDS (see Figure 2G), confirming that the photocatalytic activity of FZT Y@WDS is related to the surface area of the FZT Y@WDS Smart NPs.

### Experimental design analysis

#### Photocatalytic activity

**FIGURE 9:**
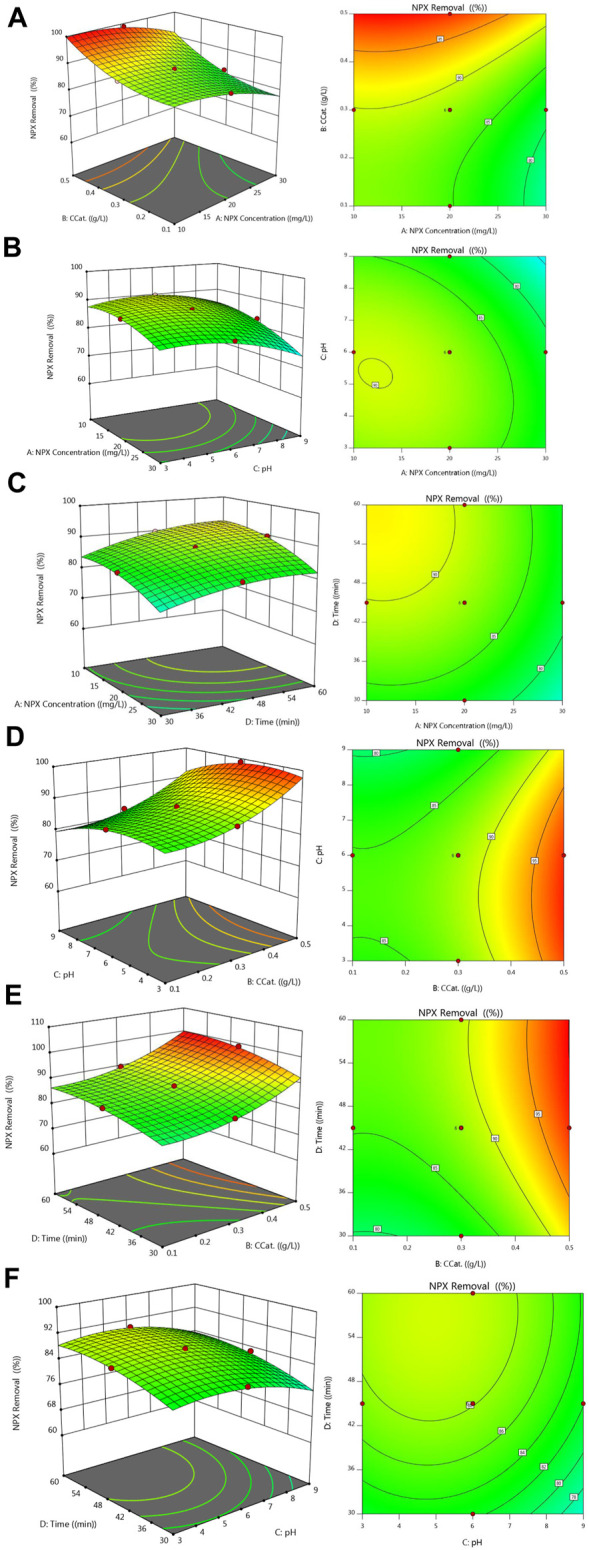
Experimental and predicted values for NAP (or NPX) removal **(A)**. C_cat_-NPX Concentration, **(B)**. pH-NPX Concentration, **(C)**. Time-NPX Concentration, **(D)**. pH-C_cat_, **(E)**. Time-C_cat_, **(F)**. Time-pH.

The interaction between factors on a given response was studied using three-dimensional diagrams. The middle variable range of such diagrams contains two factors that are changed and the other factors that are constant. NAP degradation is shown in Figure 9 as a function of pH, irradiation time, NAP contaminant concentration, and catalyst dose. The contours in Figure 9A show NAP removal as a function of catalyst loading and NAP pollutant concentration, assuming a constant value for irradiation time of 45 min and pH of 6. The contours in Figure 9B show the NAP removal as a function of pH and NAP pollutant concentration, assuming a constant value for the irradiation time of 45 min and a catalyst loading of 0.3 g/L. Contours in Figure 9C show removal NAP as a function of irradiation time and pollutant concentration NAP, assuming a constant pH of 6 and catalytic loading of 0.3 g/L. The contours plotted in Figure 9D show NAP removal as a function of pH and catalyst loading concentration, assuming a constant value for the irradiation time of 45 min and a NAP contaminant concentration of 20 mg/L. According to Figure 9E, the NAP removal depends on the catalyst loading concentration and the irradiation time, assuming a constant pH of 6 and a pollutant concentration of 20 mg/L. Based on a catalyst loading of 0.3 g/L and a concentration of NAP of 20 mg/L, Figure 9F shows the removal of NAP as a function of irradiation time and pH. Photocatalytic removal of NAP increases with increasing catalyst loading up to 0.5 g/L, as shown in Figure 9. This can be attributed to the enlargement of the active site due to higher charges, which consequently increases the absorption of a higher percentage of photons transferred to the reaction medium and reaction molecules. The photocatalytic removal of NAP increases with decreasing pH of the reaction medium and reaches its maximum value at pH = 3. There is a complex relationship between the pH of the reaction medium and the rate of photocatalytic oxidation. Figure 2 shows that the optimal pH depends on the type of pollutant as well as the PZC, which is 5.8. In the case of NAP, it should be noted that the anion NAP is formed by hydrolysis in its chemical structure. There are two different structures of NAP, which are conceivable at different pH values, both are negatively charged. Naproxen sodium is an organic sodium salt consisting of equimolar amounts of NAP (1-) anions and sodium anions. It should be noted that the pK_a_ of NAP is equal to 4.15, so that the neutral state is maintained. When the pH is above 5.9 (pH>pH_PZC_), the surface of FZT Y@WDS is negatively charged. In the presence of the anion NAP, the surface of the photocatalyst is electrostatically repelled by its negative charge. As a result, the adsorption rate of the NAP anion on the surface of the photocatalyst is reduced, which decreases the decomposition rate. Positive surface charges form on photocatalyst particles below pH_PZC_. The molecules of NAP decompose at a pH of about 3.7 (lower than pH_PZC_), resulting in a neutral state. Consequently, the adsorption of the NAP anion on the surface of the photocatalyst is enhanced by further lowering the pH to about 3 and by the formation of the NAP anion due to the dissociation of NAP and the formation of a negatively charged anion. This creates a suitable environment for the photocatalytic reaction to occur. As a result of the low pH, •HO_2_ radicals are formed form, compensating for low concentrations of •OH radicals and accelerate the degradation reactions. As a result of the low concentration of NAP, the production of OH radicals is more efficient and the recombination reaction is prevented. In addition, the reaction site is less excited, so the reactants have lower diffusion resistance. The combined effect of these two factors is to enhance the photocatalytic reaction and reduce the amount of unreacted NAP in the reaction zone.

#### PL analysis of NAP removal

**FIGURE 10:**
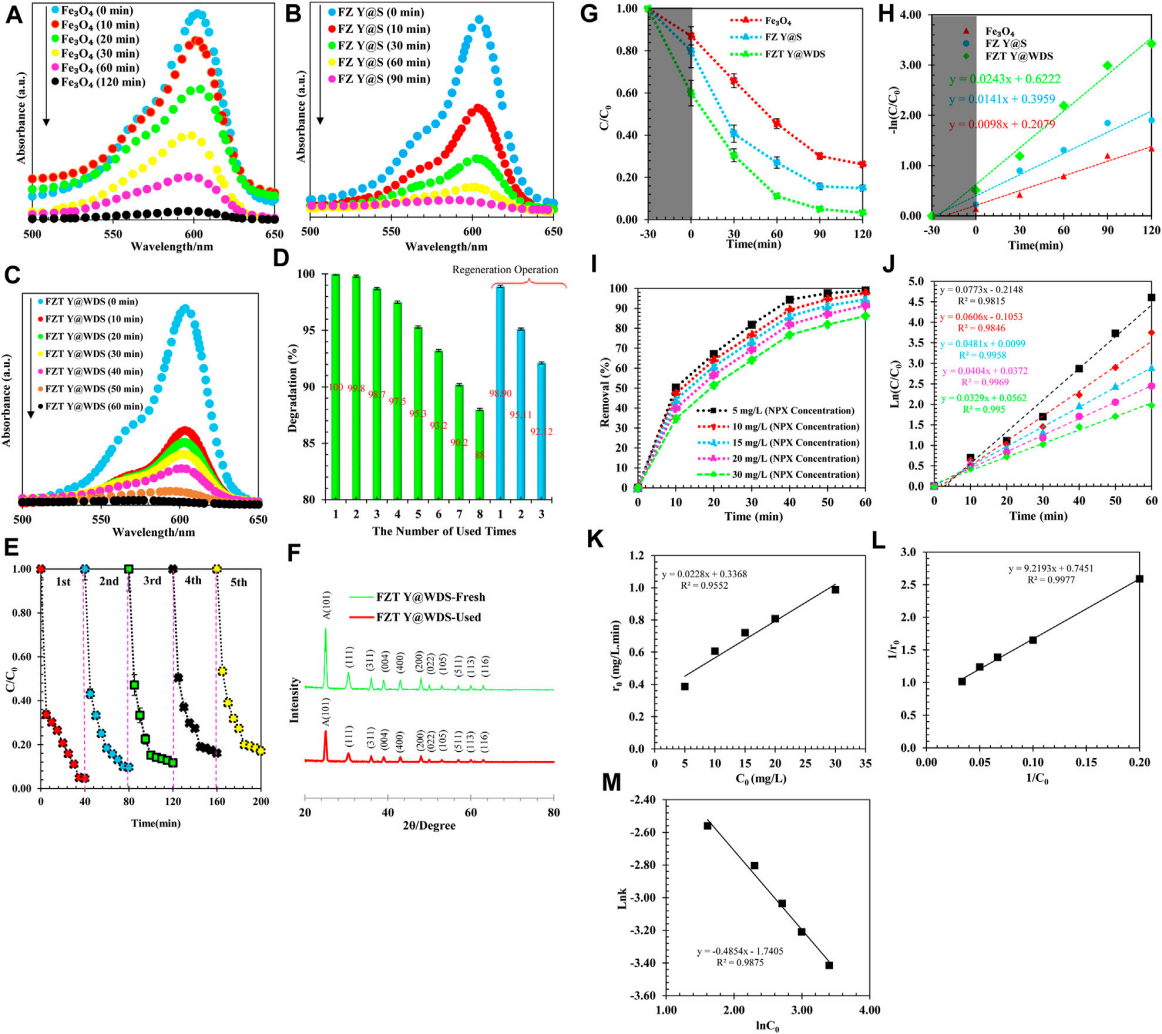
UV-Absorption spectrum for synthesized **(A)**. Fe_3_O_4_, **(B)**. FZ Y@S and **(C)**. FZT Y@WD NPs, **(D)** photocatalytic degradation of NAP by FZT Y@WDS at different times to determine the photocatalytic stability, **(E)** number of cycles of photocatalytic degradation of NAP by FZT Y@WDS under visible light irradiation (investigating the possibility of resuspended catalyst under optimal conditions), **(F)**. XRD analysis related to structural smart nanoreactors with FZT Y@WDS architecture (befor and after photocatalytic degradation of NAP), **(G)** Photocatalytic degradation of NAP under different conditions including Fe_3_O_4_, FZ Y@S and FZT Y@WDS as catalysts under visible light, **(H)** Photocatalytic kinetics study of NAP degradation under different conditions including Fe_3_O_4_, FZ Y@S and FZT Y@WDS as catalysts under visible light, **(I)** NAP removal (%) at different initial concentrations, **(J)** Photodegradation of NAP at different initial concentrations **(K)** Relationship between the initial reaction rate (r_0_) and the initial concentration (C_0_) of NAP, (L) Photocatalytic degradation of NAP by FZT Y@WDS as a L-H model **(M)** NAP photodegradation rate constant based on different initial concentrations.

During the experimental process, the photocatalytic properties of Fe_3_O_4_, FZ Y@S and FZT Y@WDS were investigated. The photocatalytic performance of the synthesized NPs was measured for comparison, and the results are shown in Figure 10. As can be seen from Figure 10A,B, the absorption of the solution NAP decreases faster in the presence of nanoreactors with FZ Y@S structure than Fe_3_O_4_ nanospheres, indicating that the nanoreactors FZ Y@S have a relatively faster degradation rate. In other words, the ZrO_2_ shell can improve the photocatalytic performance of the Fe_3_O_4_ core. When the yolk@shell nanoreactors were used, the degradation rate of NAP was improved (Figures 10B,C), and when the second TiO_2_ shell was applied to the surface of the yolk@shell FZ Y@S nanoreactors, the nanoreactors with the yolk@shell FZT Y@WDS structure were synthesized, the photocatalytic efficiency of the nanoreactors with the yolk@shell FZT Y@WDS structure increased dramatically due to the synergistic effect of the ZrO_2_ and TiO_2_ shells. Photocatalytic degradation of NAP was performed in the presence of FZT Y@WDS nanocomposite materials. Before light irradiation, mixed suspensions of NAP and FZT Y@WDS were magnetically stirred for 30 min to achieve an equilibrium between adsorption and desorption between FZT Y@WDS and NAP. The photodegradation of NAP was monitored by recording the absorption spectrum as a function of the time of light irradiation and is shown in (Figures 10A–C) (Xuan et al., 2009; Pandikumar and Ramaraj, 2012; Jia et al., 2014; Wang et al., 2014; Xu et al., 2015). The absorption intensity of NAP at 575–625 nm decreased with increasing irradiation time. This clearly shows that the concentration of NAP decreased with increasing irradiation time. During light irradiation, absorption of photons and formation of charge separations (e^−^-h^+^) occur due to the excitation of electrons (e^−^) from the valence band of Fe_3_O_4_, ZrO_2_ and TiO_2_, leaving holes in the conduction band of TiO_2_, ZrO_2_ and Fe_3_O_4_.

#### Photocatalyst separation and reusability

The recycling efficiency of catalysts has a great impact on their further use. Therefore, it is necessary to perform a rotation test for the photocatalytic degradation of the solution NAP. As shown in Figure 10D, the FZT Y@WDS nanoreactors still have good photocatalytic activity after eight cycles of degradation of the NAP solution, indicating that the prepared catalysts have excellent stability and ideal catalytic lifetime. In Figure 10D, the separation and reuse of NPs lead to a change in photocatalytic activity. After eight applications of the magnetic photocatalytic particles, the photocatalytic activity decreased slightly (Figure 10D). However, after the regeneration process, in which the catalyst is exposed to UV light in distilled water for 12 h, regeneration restores catalysts to their original state. The activity efficiency of the photocatalyst decreases after several regeneration processes. Deposition of radiation-insensitive materials or loss of NPs during recycling can lead to a reduction in photocatalytic pore activity. The recycling stability of NPs with the designed structure was evaluated using FZT Y@WDS as an example. After each photocatalytic reaction cycle, the photocatalysts were recovered by centrifugation, washed with deionized water, and transferred to a fresh NAP solution to react under the same conditions. As shown in Figure 10E, the photocatalytic activity remains almost unchanged after five consecutive recycling cycles, as the photocatalytic degradation of FZT Y@WDS still exhibits an efficiency of 87% after five cycles. As shown in Figure 10F, XRD analysis of FZT Y@WDS nanoreactors before and after the NAP degradation process confirms the high chemical stability of this nanoreactor.

#### The kinetics study of NAP degradation

In this section, the degradation efficiency of NAP from pharmaceutical wastewater was studied, and the results of pH analysis, irradiation time, photocatalyst concentration, and initial concentration of NAP were analyzed. To investigate the effects of nanoreactor composition and structure on photocatalytic performance, photodegradation experiments of NAP under simulated solar radiation (visible light) were performed. Photodegradation of NAP was carried out using the FZT Y@WDS nanoreactor under visible light irradiation (λ> 420 nm) to evaluate its photocatalytic activity. The FZT Y@WDS nanoreactor samples were mixed with a certain amount of NAP solution and stirred in the dark for 0.5 h to reach absorption equilibrium. The change in the concentration of NAP during the photodegradation process is shown in Figure 10G,H. It was found that about 41% of NAP molecules are adsorbed on the surface of the nanoreactor when the absorption reaches equilibrium in the dark, while 21% and 13% of NAP molecules are absorbed on FZ Y@S nanoreactor and Fe_3_O_4_ NPs, respectively. After 4 h of visible light irradiation, 97% of the compound NAP was almost completely degraded by the FZT Y@WDS nanoreactor, which was higher than the degradation observed with the FZ Y@S nanoreactor (86%) and the Fe_3_O_4_ nanophotocatalyst (74%). As shown in Figure 10G, the second TiO_2_ shell increases the photocatalytic rate of FZT Y@WDS. From Figure 10G, it can be seen that the removal of the impurity NAP has an optimum value because the impurity NAP can also act as a recombination center of light-induced charge carriers and decrease the crystallinity of FZT Y@WDS, FZ Y@S and Fe_3_O_4_, leading to a decrease in the photocatalytic activity of the photocatalysts. In addition, the Y@WDS has a significant effect on the light-based activity of the catalyst. The natural logarithm of absorption as a function of irradiation time is shown in Figure 10H. It is obvious that the degradation process follows pseudo-first order kinetics according to the research (Kim et al., 2012). As can be seen from the results, the degradation rates by the nanophotocatalysts Fe_3_O_4_, FZ Y@S, and FZT Y@WDS are 0.588 h^−1^, 0.846 h^−1^, and 1.458 h^−1^, respectively. The contribution of the ZrO_2_ and TiO_2_ compounds synthesized as shells on the Fe_3_O_4_ yolk to the degradation rate is 0.258 h^−1^ and 0.612 h^−1^, respectively. It can be concluded that both the ZrO_2_ and TiO_2_ shells significantly enhance the photocatalytic activity of Fe_3_O_4_ under visible light, and that there is a synergistic effect apparently due to the coexistence of the two compounds ZrO_2_ and TiO_2_ on the Fe_3_O_4_ core. The performance of the synthesized photocatalysts with different structural architecture under visible light irradiation determines their degradation rate as follows:

## FZT Y@WDS > FZ Y@S > Fe_3_O_4_

This result shows that the type of Y@WDS can significantly increase the degradation rate. Physical adsorption and chemical reactions are the main phases of the removal of NAP with the heterogeneous photocatalyst FZT Y@WDS. The Langmuir–Hinshelwood kinetic model (LH) was used to describe the photocatalytic degradation rate of NAP by plotting ln (C_0_/C) *versus* time (t) at different concentrations (Eq. 9) (Kumar et al., 2008):

$$\ln\frac{C_{i}}{C}=k.t$$ (9)

Where C_i_(mg/L) is the initial concentration of NAP and C (mg/L) is the concentration at time (t) after irradiation, and k is a first-order pseudo-rate constant. As shown in Figure 10I, the first-order pseudo-rate constant was determined based on a linear slope. According to the results of Figure 10J, the maximum reaction rate at an initial concentration of 5 mg/L NAP is 0.0773 min^-1^, which is about 2 times higher than the reaction rate at an initial concentration of 30 mg/L NAP. According to the results shown in Figure 10J, the reaction rate decreases at higher concentrations. The NAP absorbed amount qe (mg/g) of FZT Y@WDS nanocomposite was calculated using the following Eq. 10:

$$q_{e}=\frac{\left(C_{i}-C_{e}\right).V}{m}$$ (10)

​Where C_i_ and C_e_ are the initial and equilibrium concentrations, respectively, of NAP (mg/L). V is the volume of NAP solution (mL) and m is the mass of FZT Y@WDS nanocomposite (mg). the NAP adsorption (%) was calculated using the following equation (Eq. 11):

$$Adsorption\;\left(\%\right)=\frac{\left(C_{i}-C_{e}\right).V}{m}$$ (11)

In Figures 10K-M, the initial photodegradation rate (r_0_) gradually increased from 0.39 mg/L to 0.99 mg/L when the concentration of NAP was increased from 5 mg/L to 30 mg/L. The FZT Y@WDS nanoreactor undergoes photocatalytic degradation of NAP on its surface, with the photodegradation rate increasing with increasing adsorption. The surface coverage of the FZT Y@WDS nanoreactor increases with increasing concentration of NAP molecules on its surface. By increasing the electron transfer efficiency, the charge generated by the light and the electron transfer efficiency of the NAP molecules increase, increasing the photodegradation rate (r_0_). Therefore, an increase in the adsorbed surface area and the charge generated by the light leads to an increase in the electron transfer efficiency of the NAP molecules, increasing the photodegradation rate (r_0_). It was found that the kinetic constant of NAP photodegradation gradually decreases (from 0.0773 to 0.0329 min^-1^) and the correlation coefficient increases (from 0.9815 to 0.995) (at different initial concentration). According to the following experimental formula (Eq.13), the reaction rate (k) and the substrate concentration are generally correlated:

$$k=a{\left[NAP\right]}^{n}$$ (12)

$$\ln\;k=\ln\;a+nln\left[NAP\right]$$ (13)

In this equation, n represents the correlation index, and [NAP] denotes the initial concentration (NAP) (mg. L^-1^). Based on the range of initial concentrations used in the experiments, linear regression was used to determine the relationship between the kinetic constant of NAP photodegradation and its initial concentration (5–30 mg/L). As shown in Figure 10 (L), the reaction rate k is related to the concentration NAP as follows:

$$\ln\;k=\ln\;0.0196+0.2074.\ln\left[NAP\right]\left(R2=0.9977\right)$$ (14)

The reaction mechanism for NAP degradation can be illustrated as the following Eqs. 15–19:

$${Photon}^{*}+FZT\;Y@WDS\;\to h^{+}+FZT\;Y@WDS\;\left(e^{-}\right)$$ (15)

$$FZT\;Y@WDS\;\left(e^{-}\right)+O_{2}\to \cdot O^{2-}$$ (16)

$$O^{2-}+H^{+}\to \cdot OH$$ (17)

$$H_{2}O+h^{+}\to \cdot OH+H^{+}$$ (18)

$$NAP+\cdot O^{2-}/•OH/h^{+}\;\to degradation\;products$$ (19)

**FIGURE 11:**
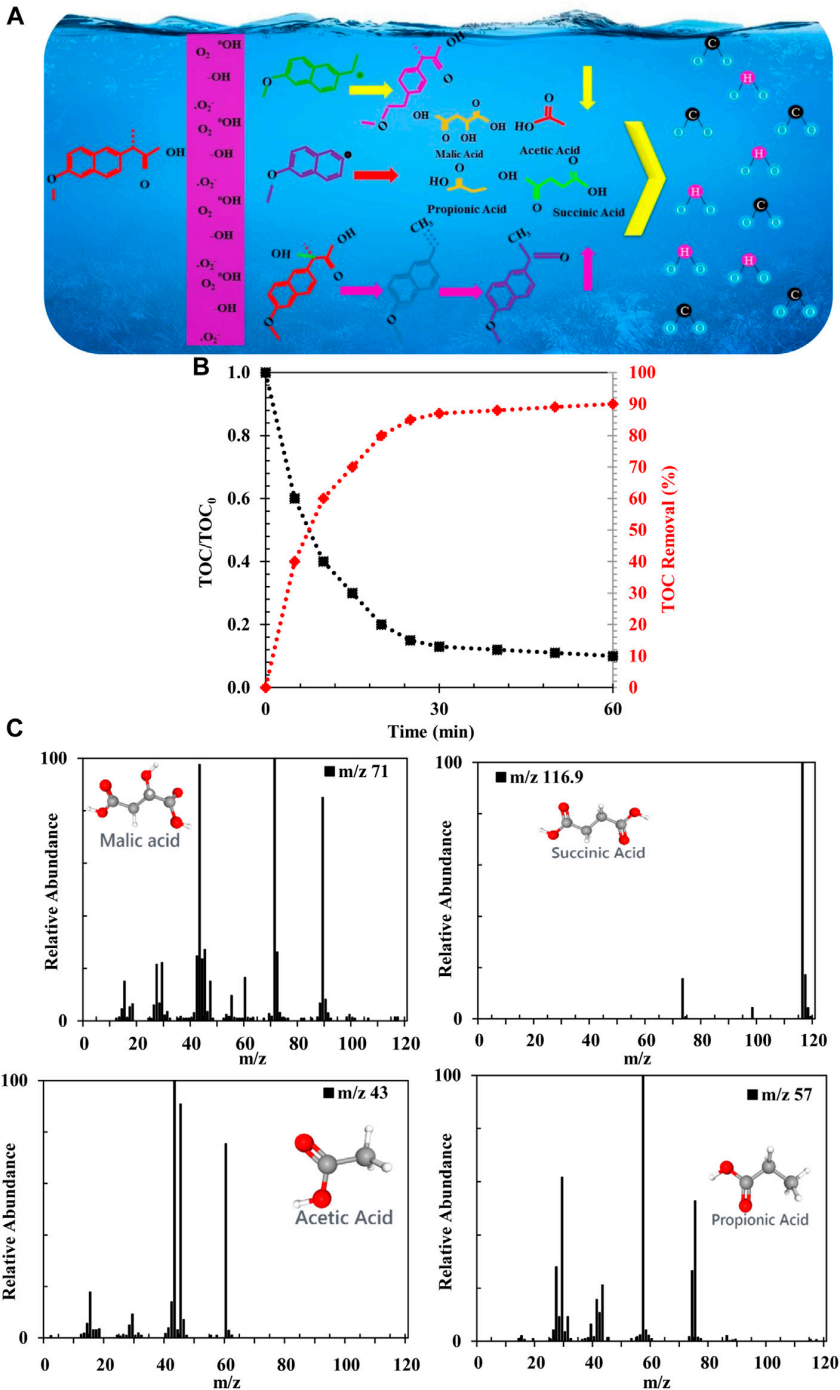
**(A)** Schematic of photocatalytic process for NAP degradation on the FZT Y@WDS architectural Photocatalyst. **(B)** Photocatalytic activity of the NAP solution as a function of total organic carbon (TOC), **(C)** GC-MS analysis to identify intermediate products of NAP degradation using FZT Y@WDS nanoreactors.

According to the results, NAP and its degradation intermediates can be further degraded by reactive species, ultimately leading to ring openings and CO_2_ and H_2_O oxidation. Based on the identification of intermediates and mineralization results, possible degradation pathways of NAP are shown in Figure 11A. NAP is decarboxylated and hydroxylated by FZT Y@WDS mainly via three pathways (Fan et al., 2019). According to Figure 11A, there are three pathways of oxidative degradation. Naphthalene ring interactions with •OH are electrophilic additive interactions that initiate degradation of NAP in oxidation pathway I (Arany et al., 2013; Dulova et al., 2017; Chi et al., 2019). The II and III pathways generate carbon-based radicals by oxidation of NAP with h^+^ and •O^2-^ (Wang et al., 2018). To confirm the mineralization of the pollutant solution, total organic carbon (TOC) was investigated during photolysis of NAP at ambient temperature and neutral pH. A decrease in TOC content with increasing light irradiation time was observed (Figure 11B), indicating the mineralization of NAP by the architectural photocatalyst FZT Y@WDS under visible light. A performance of 91% was observed after 60 min of light irradiation (Figure 11B). The TOC result confirmed the oxidation of NAP to CO_2_, H_2_O and some small molecules. In this process, the intermediates formed in the ring-opening reaction decompose into acetic acid, malic acid, succinic acid, and propionic acid, and then mineralize to CO_2_ and H_2_O (Jallouli et al., 2016; Gao et al., 2017; Fan et al., 2019).

### Identification and characterization of intermediate products of NAP

The study of NAP intermediates resulting from degradation in purified NAP solution was performed using FZT Y@WDS nanoreactors under visible light irradiation. The chromatogram of the decomposed solution showed the peaks corresponding to the four by-products succinic acid, malic acid, acetic acid and propionic acid. The main m/z signals of the detected ions for each peak in the experiments, the proposed structures for the side products and their spectra are shown in Figure 11C.

### Photocatalytic mechanism model

**FIGURE 12:**
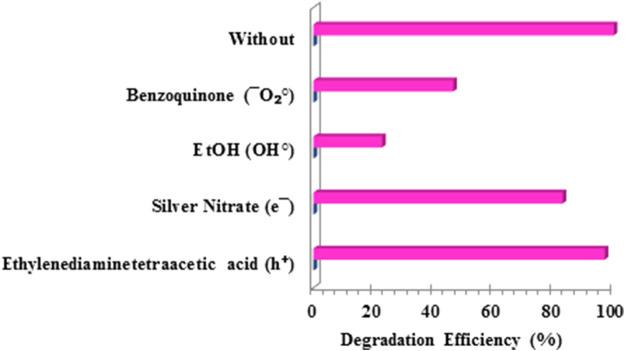
Efficiency of naproxen degradation by the photocatalyst FZT Y@ DS under optimal conditions and in the presence of charge carriers.

In order to determine the mechanism of degradation of the pollutant NAP by the photocatalyst FZT Y@ DS, experiments were performed with charge carriers to capture the active species of the photocatalytic reactions. For this purpose, EDTA (for holes), silver nitrate (for electrons), ethanol (for OH^**•**^) and benzoquinone (for $O_{2}^{-}$ ) were used as trapping agents (Ghorai et al., 2021; Yang et al., 2021). As shown in Figure 12, the addition of silver nitrate has a negligible effect on the degradation efficiency of NAP, indicating the negligible role of electrons in the photochemical degradation of the pollutant naproxen. In contrast, the addition of EDTA, benzoquinone, and ethanol decreased the yield, indicating that the cavity radicals, $O_{2}^{-}$ and OH^**•**^, play a significant role in the photodegradation process of NAP.

Electron charge transfer is affected by the position of the conduction band (CB) and the valence band (VB) determined for the photocatalysts Fe_3_O_4_, ZrO_2_, and TiO_2_ according to Eq’s (20) and (21) (Ojha et al., 2019):

$$E_{CB}=X-E_{0}-\frac{1}{2}E_{g}$$ (20)

$$E_{VB}=E_{g}+E_{CB}$$ (21)

where in the above relations $E_{CB}$ , $E_{VB}$ and $E_{g}$ represent the conductivity potential, the capacitance potential and the energy band gap, $E_{0}=4.5\;eV$ is constant for all photocatalysts and represents the energy of free electrons, moreover X is the absolute electronegativity and for Fe_3_O_4_, ZrO_2_ and TiO_2_ the sequence has values of 5.73, 5.91 and 5.81, respectively (Bi et al., 2020b; Jiang et al., 2021; Pham et al., 2021). $E_{g}$ of each photocatalyst was also determined by UV-vis DRS analysis.

**FIGURE 13:**
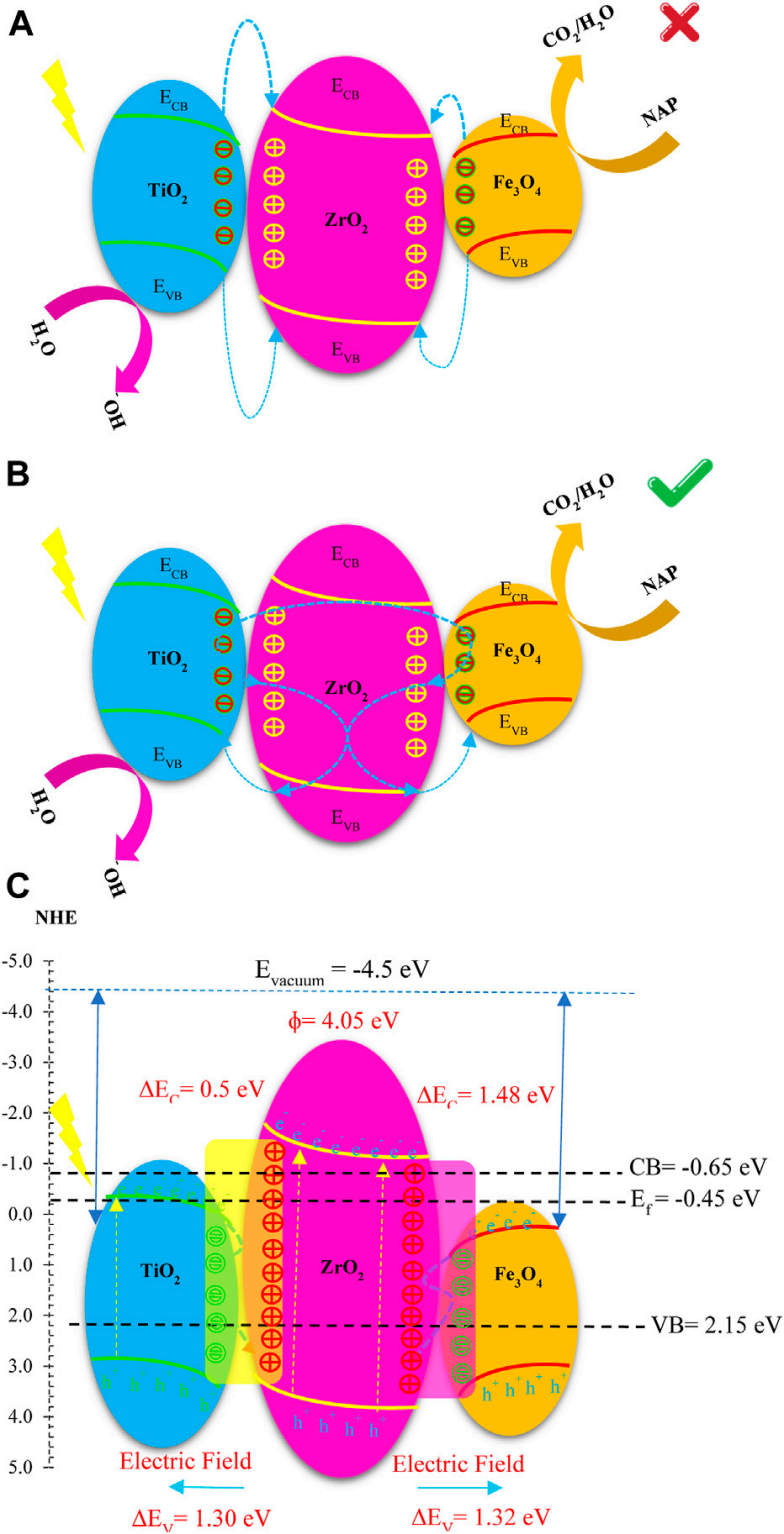
Electron transfer path in FZT Y@ DS nanoreactor **(A)** Compatible **(B)** Incompatible, **(C)** photogenerated charge carrier transfer process in S-scheme mode.

There are two possible mechanisms for the degradation of NAP using the ternary nanoreactor FZT Y@ DS under light irradiation, as shown in Figure 13. Under light irradiation, the electron-hole pairs of Fe_3_O_4_, ZrO_2_ and TiO_2_ were excited in the conduction and capacitance layers, respectively. If it is suggested by the mechanism model of Figure 13A that the electron is transferred from the conducting layers of Fe_3_O_4_ and TiO_2_ to the conducting layer of ZrO_2_, this contradicts and violates the laws of thermodynamics. According to the obtained results, a double S-scheme structure is proposed to improve the degradation efficiency of the pollutant NAP by the nanoreactor FZT Y@ DS. As shown in Figure 13B, the electrons generated by the ZrO_2_ conductive layer are directed to the Fe_3_O_4_ and TiO_2_ conductive layers because the electrons in both samples have the same charge. The accumulation of electrons towards the ZrO_2_ conductive layer creates holes in this layer and the electrons are sent towards the Fe_3_O_4_ and TiO_2_ conductive layers. This S-scheme structure improves the charge transfer efficiency and lifetime by reducing the recombination of electrons and holes, as fully shown in Figure 13C.

## Conclusion

Structural NPs of FZT Y@WDS yolk@shell were prepared by a fabrication process consisting of calcination, selective, sol-gel, solvothermal etching and methods. The results of FESEM, XRD, EDS, XPS, BET, TEM, PZC, electrochemical impedance spectroscopy, Matt-Schottky, DRS, polarization, Tafel, CV and UPS analyzes confirmed the successful formation of smart nanoreactors with FZT Y@WDS architecture and very favorable physical properties. In this research, we investigated the photocatalytic performance of FZT Y@WDS yolk@shell structured NPs using response surface method. The calcination temperature and photocatalytic performance are directly influenced by the parameters of catalyst concentration, pH of reaction medium, irradiation time and pollutant concentration. The results show that all the factors studied are effective and the FZT Y@WDS structured NPs are a very suitable photocatalyst that can operate in the visible light range, providing an alternative for wastewater treatment and reducing environmental impact. The advantages of FZT Y@WDS yolk@shell structured NPs as nanoreactors can be summarized as follows: (1). Physical and chemical properties that can be manipulated (adjustable) of core@shell; (2). A moving core within the shell to catalyze collaborative efforts (involves mutual assistance in working toward a common goal). (3). A homogeneous reaction medium for heterogeneous catalysis because the cavities act like a reservoir. (4). Protection of the catalytic core NPs and prevention of condensation. and (5). Controllable penetration and diffusion rates.

## Data Availability

The original contributions presented in the study are included in the article/Supplementary Material, further inquiries can be directed to the corresponding authors.
