# Corrigendum: Yolk@Wrinkled-double shell smart nanoreactors: new platforms for mineralization of pharmaceutical wastewater

**DOI:** 10.3389/fchem.2024.1385825

**Published:** 2024-03-06

**Authors:** Masoud Habibi Zare, Arjomand Mehrabani-Zeinabad

**Affiliations:** Department of Chemical Engineering, Isfahan University of Technology, Isfahan, Iran

**Keywords:** structural nanoreactor, heterojunction, visible light irradiation, yolk@shell structure, smart nano particles

In the published article, there was an error in Figure 1B as published. The incorrect version of the figure was mistakenly uploaded due to an oversight. The corrected Figure 1B and its caption appear below.

**FIGURE 1:**
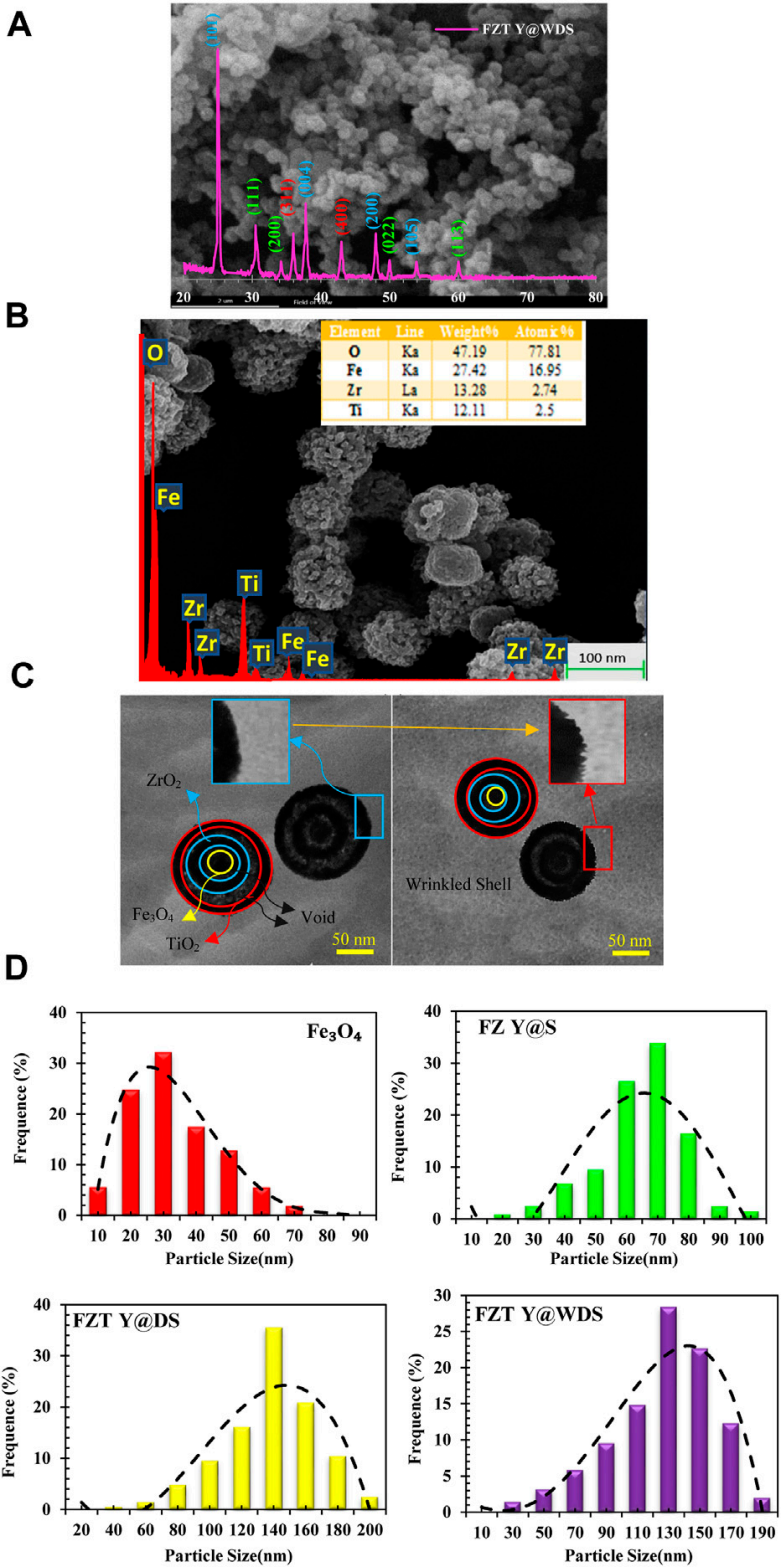
**(A)** XRD, **(B)**. SEM and EDS analysis **(C)**. TEM images related to structural smart nanoreactors with FZT Y@WDS architecture, **(D)**. The average sizes of Fe3O4, FZ Y@S, FZT Y@DS and FZT Y@WDS NPs.

The authors apologize for this error and state that this does not change the scientific conclusions of the article in any way. The original article has been updated.

