# Supplementary material for: Yolk@Wrinkled-double shell smart nanoreactors: new platforms for mineralization of pharmaceutical wastewater
Source: Front Chem. 2023 Jun 6;11:1211503. doi: 10.3389/fchem.2023.1211503 (PMC10281210; doi:10.3389/fchem.2023.1211503)
Supplement: Supplementary file 1 [file DataSheet1.docx]

Supplementary Materials for

**Yolk@Wrinkled-Double Shell Smart Nanoreactors: New Platforms for Mineralization of** **Pharmaceutical Wastewater**

Masoud Habibi Zare^1*^, Arjomand Mehrabani-Zeinabad^1*^

^1^Department of Chemical Engineering, Isfahan University of Technology, 84156-83111 Isfahan, Iran

^*^ Corresponding authors: E-mail: [masoud.habibi@ce.iut.ac.ir](mailto:masoud.habibi@ce.iut.ac.ir)

**Table of Contents**

- Experimental Section
- Experimental Materials
- Synthesis Procedure of FZT Y@DS Smart Nanoreactors
- Determination of Point of Zero Charge (PZC)
- Reusability and Recovery of Photocatalysts
- Electrochemical Impedance Spectroscopy (EIS)
- Mott-Schottky Plot Analysis
- Cyclic Voltammetry Diagrams
- Experimental Design of NAP Degradation
- Photocatalytic Activity
- Experimental Design Analysis
- Accuracy of the Developed Model
- Effect of Process Parameters on NAP Degradation
- Optimization and Validation of NAP Removal
- **Experimental Section**

Subsequently, the smart nanoreactors with FZT Y@WDS architecture were characterized by FESEM, XRD, EDS, XPS, BET, TEM, PZC, electrochemical impedance spectroscopy, Matt-Schottky, DRS, polarization, Tafel, CV and UPS analyzes. The lack of research data on the role of the different parameters of the initial concentration of NAP, the initial pH of the solution, the amount of charged photocatalyst and the duration of irradiation on the morphology and the rate of photocatalytic degradation of NAP using smart nanoreactors with FZT Y@WDS architecture is one of the motivating factors for conducting this research. The analysis of this activity was carried out using the RSM method of design of experiments with Design Expert 11 software.

- **Experimental Materials**

All materials were purchased from Sigma-Aldrich and Merck Companies and used as supplied. Double-distilled water has an electrical resistance value of 18.2 MΩ.cm^-1^ (at 25 °C).

**Table S1:** Physical and chemical properties of NAP.

| Chemical Formula | Log K_ow_ | Melting point | Molecular Weight [g/mol] | Solubility  [mg/L at 25 °C] | pK_a_ | Molecular Structure |
| --- | --- | --- | --- | --- | --- | --- |
| C_14_H_13_NaO_3_  Purity (%) =98 | 3.18 | 152-154 °C | 230.26 | 15.9 | 4.15 | 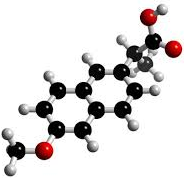 |

- **Synthesis Procedure of FZT Y@DS Smart Nanoreactors**

1.03 gr FeCl_3_.6H_2_O and 0.15 gr trisodium citrate (Na_3_C_6_H_5_O_7_) were dissolved in 50 mL ethylene glycol (C_₂_H_₆_O_₂_-EG) to synthesize Fe_3_O_4_ NPs by a solvothermal process. Then, a solution of 50 mL EG, containing 4 gr of NaAc, was added to the previous solution. The mixture was heated at a temperature of 200 $℃$ for 10 hr, transferred to a Teflon-lined stainless-steel autoclave with a capacity of 150 mL, cooled to room temperature, washed with alcohol and dried. Next, 0.1 gr of Fe_3_O_4_ NPs were dispersed in a mixture of ethanol and DI water using ultrasonication. Then, 5 mL of concentrated ammonium hydroxide solution was added under the ultrasonic method. Afterwards, 0.5 mL TEOS was added dropwise to the solution, and after 45 min, another 0.5 mL TEOS was added. After washing with ethanol, the product is dispersed in ethanol and forms a colloidal dispersion. 15 mL of the colloidal Fe_3_O_4_@SiO_2_ dispersion was added to the solution, 0.05 gr of hydroxypropyl cellulose (HPC), ethanol (95 mL) and DI water (0.5 mL) with mechanical stirring. Then, 1.90 mL of zirconium (IV) butoxide solution diluted with ethanol was added dropwise. The product obtained was washed with ethanol after standing overnight at room temperature and dried. The organics were removed and the ZrO_2_ shell crystallized by heating to 500 °C under air atmosphere. The partial etching process to remove the SiO_2_ shell was performed by dispersing Fe_3_O_4_@SiO_2_@ZrO_2_ NPs in a 100 mL NaOH solution (0.5 M) at a temperature of 60 °C for 12 hr. This step was continued for 24 hr with 100 mL of new NaOH solution, and the obtained product was washed with DI water and dried. The calcination procedure was carried out as before. For the synthesis of Fe_3_O_4_@ZrO_2_@SiO_2_, 5 mL of ethanol solution from Fe_3_O_4_@ZrO_2_ nanoreactors (FZ Y@S NPs) was added to 80 mL of ethanol, 20 mL of deionized water and 5 mL of concentrated ammonia solution (28 wt%). After sonication, 0.5 mL of TEOS was added dropwise, and the reaction was carried out at room temperature with mechanical stirring for one hour. The obtained products were washed with DI water and ethanol and dried. To synthesize Fe_3_O_4_@ZrO_2_@SiO_2_@TiO_2_ NPs with a yolk@shell structure, 0.08 gr of Fe_3_O_4_@ZrO_2_@SiO_2_ nanospheres were first dispersed in concentrated ethanol and ammonia solution (28 wt%) under ultrasonication. The reaction was carried out for 24 hr at 45 ^°^C with constant mechanical stirring, adding 0.5 mL of TIPO dropwise. The obtained product was washed with DI water and ethanol and then dried. Removal of the organic material and crystallization of the TiO_2_ shell by calcination were carried out as before. For the final product and synthesis of FZT Y@DS NPs smart nanoreactors, the partial etching process was performed as before. By partial etching process using alkaline hydrothermal process, the synthesis of structural smart nanoreactors Yolk@Wrinkled-Shell (FZT Y@WDS) with wrinkled shell was carried out. Under ultrasonication, 0.05 gr of structural smart nanoreactors FZT Y@DS was dispersed in 10 mL of aqueous 0.1 M sodium hydroxide solution. Then, the suspension solution was placed in a stainless-steel reactor with Teflon coating (capacity 100 mL). The reactor was heated to a temperature of 200 °C for 10 hr, and then the separation was dried. Then the sediment was immersed in 150 mL of dilute hydrochloric acid solution of 0.1 M, washed with deionized water and dried to finally synthesize FZT Y@DS structural smart nanoreactors with a unique yolk and wrinkled shell architecture (As seen in Figure S1).


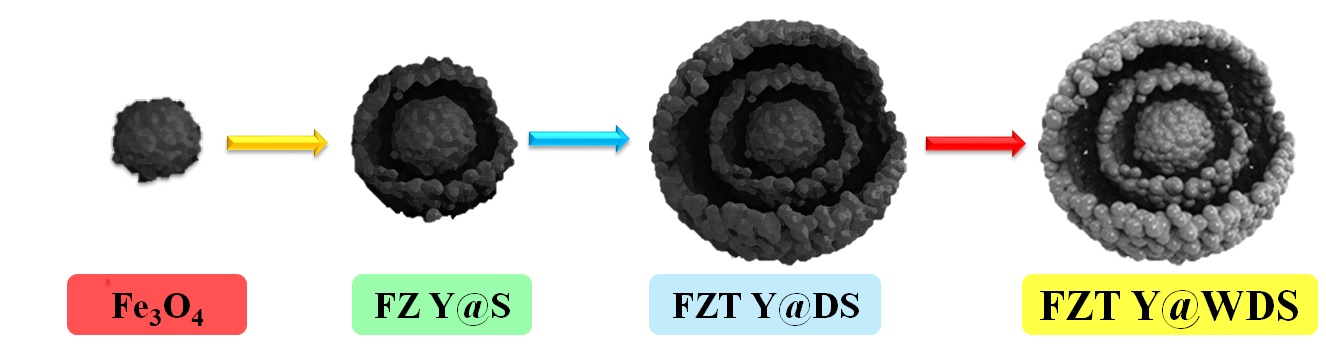


**Fig. S1:** Schematic illustration of Synthesis Steps of FZT Y@WDS Smart Nanoreactors

- **Determination of Point of Zero Charge (PZC)**

To determine the point of zero charge (PZC) of FZT Y@DS structural smart nanoreactors, the salt addition method was used[1] . By adding 0. 5 gr of Y@DS FZT structural smart nanoreactors to solutions with the same ionic strength but different pH values, the point of zero charge was determined. The synthesized FZT Y@DS structural smart nanoreactors were added to 50.0 mL of a NaNO_3_ solution (0.1 M). Sulfuric acid (0.1 M) and sodium hydroxide (0.1 M) solutions were used to adjust the pH different values. The pH of each solution was measured in a shaking incubator for one day before and after 24 hr of mixing, and the values are expressed as pH_i_ and pH_f_, respectively.

- **Reusability and Recovery of Photocatalysts**

Magnetic separation of FZT Y@WDS structural smart nanoreactors was performed in this study using a 1.3 Tesla magnet. In the photocatalytic process, the separated NPs were re-used. The photocatalytic degradation process was carried out in six steps, followed by the photocatalyst recovery process. This was achieved by immersing the NPs in distilled water and exposing them to visible light for 24 hr. After recovery, the photocatalytic activity was re-evaluated.

- **Electrochemical Impedance Spectroscopy (EIS)**

We used a PalmSens3 potentiostat/galvanostat/impedance analyzer to perform EIS measurements. Throughout this study, platinum wire and Ag/AgCl were used as auxiliary electrodes, Ag/AgCl as reference electrodes, and modified mild steel plates as working electrodes. By joining a copper wire with a mild steel plate (10×10×0.5 mm3) using conductive silver adhesive, a working electrode could be constructed. There was approximately 100 mm2 of exposed surface area, which was embedded in polyester resin. Pigments were added to the epoxy resin at a concentration of 10 wt% and ground. Then the appropriate amount of polyamide hardener was added to the epoxy nanocomposite. The epoxy coating was applied to the steel sheet using an adjustable film applicator. The dry thickness of the coating was about 200 ± 5 μm. The electrochemical properties of steel samples immersed in 3.5 wt% NaCl solutions without and with nanopigments were studied. The EIS measurement was performed by applying a sinusoidal potential of 10 mV over the frequency range from 50 kHz to 0.1 Hz. A suspension was prepared from Fe3O4, FZ Y@S, FZT Y@DS, and FZT Y@WDS samples in four separate chambers, which was then coated on FTO glass by immersion method. An electrochemical cell for electrochemical impedance spectroscopy was prepared and investigated from Fe3O4, FZ Y@S, FZT Y@DS, and FZT Y@WDS samples. Electrochemical impedance spectroscopy (EIS) of Research Princeton Applied Model 2273 PARSTAT was used to check the electrical conductivity.

- **Mott-Schottky Plot Analysis**

Photocatalysts that share the same energy band gap behave differently because their position in the minimum CB (CBM) and maximum VB (VBM) oxidation-reduction potentials determines their oxidation-reduction ability. In this report, the Mott-Schottky technique was used to determine the CB position in the redox potential value. According to Eq. (S1), the apparent capacitance is calculated by the Mott-Schottky method as a function of the potential. The CBM may be calculated from C=0 by extrapolating. Based on the reference electrode and the conducting medium, the CBM value is determined. However, in reports, the CBM level should be converted using Eq. (S2) so that it can be compared with different electrode systems and conductive media (electrolyte) to ensure consistency. The CBM versus the NHE can be determined by combining this result with the bandgap energy obtained from DRS analysis, the CBM can be determined in relation to the NHE. A Nernst equation (Eq. (S3)) was used to investigate the oxidation-reduction potential of various species and their pH changes. For water splitting, photocatalysts with a CBM of less than -0.41 V vs. NHE (E 2H^+^/H_2_, pH = 7) facilitate the reduction of H_2_O to H_2_, while photocatalysts with a VBM higher than 0.82 V vs. NHE (E O_2_/H_2_O, pH = 7) facilitate the oxidation of H_2_O to O_2_. For environmental applications, photocatalysts with a CBM of less than -0.78 V vs. NHE (E ^•^OH/OH^-^, pH = 7) and VBM higher than 2.28 V vs. NHE (E ^•^OH/OH^-^, pH = 7) are preferred because they can generate O^2•^ and OH^•^ radicals to degrade pollutants. Additionally, the simplest approach assumes that the CB and VB of the heterojunction photocatalysts are shifted in opposite directions by the same magnitude.

| $\frac{1}{C_{SC}^{2}}=\frac{2}{e\varepsilon\varepsilon_{0}N_{A}^{2}}\left( E-E_{FB}-\frac{kT}{e} \right)$ | (S1) |
| --- | --- |
| $E^{0}=E_{NHE}^{25℃, pH=0}=E+c$ | (S2) |
| $E^{pH}=E^{0}+0.059\times pH$ | (S3) |

where C_SC_ is the baroelectric capacitance of the space between the electrodes, ε is the dielectric constant of the reaction, e is the electron charge (F.cm^-2^), ε_0_ is the dielectric constant of the vacuum, N is the number of denominators, k is the Boltzmann constant, T is the experimental temperature (K), and A is the surface area of the electrode in contact with the electrolyte (cm). E is the applied potential (V). E_FB_ is estimated by extrapolating a linear fit of the MS plot to obtain the x-axis intercept (V). The values of c were given in previous studies (kT/e is approximately 0.0257 V at 25 ^°^C)[2-7].

- **Cyclic Voltammetry Diagrams**

To perform all electrochemical measurements, 5 mg of catalyst (one sample each of Fe_3_O_4_, FZ Y@S, FZT Y@DS, and FZT Y@WDS) and 100 µL of Nafion solution (5 wt%) were dispersed in 5 mL of H_2_O/EtOH solution (volume/volume ratio 4:1 (v/v)) for 30 min to form a homogeneous suspension (catalyst ink). Then, 15 μL of the catalyst ink was poured onto a 5 mm diameter glassy carbon electrode (surface area = 8.50 mm^2^) as a working electrode with a constant mass loading of 0.15 mg/cm^2^. A linear reciprocating voltammogram (from 0 to -0.5 V with a scan rate of 1 mV/s) in sulfuric acid (0.5 M) using a standard calomel electrode and platinum wire as the reference electrode and an auxiliary electrode (counter electrode). was measured Pt/C (20 wt% platinum on graphitic carbon) was obtained commercially from Sigma-Aldrich.

An electrochemical study in phosphate buffer solution at pH$\cong$7 was used to construct a Mott-Schottky diagram based on electrochemical data. Graphite and Ag/AgCl (in 3 M NaCl solution) electrodes were used as reference and auxiliary electrodes, respectively. Electrochemical station data were collected using a range of applied potentials (from -0.1 to 0.1 V) at 1000 Hz on the capacitance of the space charge layer. In accordance with Eq. (S4), the potential of the reversible hydrogen electrode (RHE) was recorded.

| E (vs. RHE) =E (vs. Ag/ AgCl) + 0.197 V + 0.0592 × pH. | (S4) |
| --- | --- |

- **Experimental Design of NAP Degradation**

This study investigates the effects of operating parameters, including concentration NAP, photocatalyst dose, pH, and time on the process optimization by using an experimental design approach (Box-Benken design (BBD) in three levels with α=1 (center level) was used). Table S2 shows the variables and their respective ranges for photocatalytic degradation.

**Table S2:** The variables and their coded and uncoded values in real and coded values for the experimental design

| Symbol | Factor | Code Levels of Variables | | |
| --- | --- | --- | --- | --- |
|  |  | **-1** | **0** | **1** |
| X1 | NAP Concentration (mg/L) | 10 | 20 | 30 |
| X2 | C_Cat_. (g/L) | 0.1 | 0.3 | 0.5 |
| X3 | pH | 3 | 6 | 9 |
| X4 | Time (min) | 30 | 45 | 60 |

- **Experimental Design and Optimization**

RSM was used to prepare the model and analyze the results (Table S3). The effects of four potentially important parameters on the photocatalytic performance of FZT Y@WDS smart NPs were studied, and the conditions leading to maximum catalytic activity were determined by optimizing the response surface. RSM was used in combination with the BBD method. The response (y), i.e., the removal of NAP (NPXR), and the corresponding response surface can be expressed as Eq. (S5) because all independent parameters ($x_{i}$), i.e., NAP concentration, solution pH, the amount of photocatalyst loaded, and irradiation time, are measurable. It is assumed that these parameters are continuous and controllable with negligible errors:

| (S5) | $y=f(x_{1},x_{2},x_{3},x_{4})$ |
| --- | --- |

Other factors such as temperature and light intensity are assumed to be constant. To avoid systematic errors, experiments were performed randomly, and the average of three replicates was reported as the removal efficiency of NAP.

**Table S3:** Design matrix for test factors and NAP degradation responses at different factor levels.

| Run | Factor 1 | Factor 2 | Factor 3 | Factor 4 | Response | |
| --- | --- | --- | --- | --- | --- | --- |
|  | **A: NAP Concentration (mg/L)** | **B: C_Cat._ (g/L)** | **C: pH** | **D: Time (min)** | **NAP Removal (%)** | |
|  |  |  |  |  | **Actual** | **Predicted** |
| 1 | 20 | 0.3 | 6 | 45 | 88 | 88.09 |
| 2 | 20 | 0.3 | 3 | 45 | 87.3 | 88.09 |
| 3 | 30 | 0.1 | 9 | 30 | 66 | 66.25 |
| 4 | 20 | 0.3 | 6 | 45 | 87.6 | 87.52 |
| 5 | 20 | 0.3 | 6 | 45 | 87.5 | 88.09 |
| 6 | 20 | 0.3 | 6 | 45 | 87.3 | 87.68 |
| 7 | 20 | 0.3 | 6 | 45 | 88.2 | 88.09 |
| 8 | 30 | 0.1 | 3 | 30 | 73.5 | 73.95 |
| 9 | 20 | 0.1 | 6 | 45 | 85.5 | 85.49 |
| 10 | 30 | 0.5 | 3 | 30 | 87.7 | 88.09 |
| 11 | 10 | 0.1 | 3 | 60 | 85.9 | 86.47 |
| 12 | 20 | 0.3 | 9 | 45 | 82.8 | 82.42 |
| 13 | 10 | 0.1 | 9 | 60 | 85.3 | 85.22 |
| 14 | 30 | 0.5 | 9 | 60 | 85.2 | 85.44 |
| 15 | 30 | 0.1 | 9 | 60 | 71.9 | 72.02 |
| 16 | 10 | 0.5 | 3 | 30 | 90.9 | 91.23 |
| 17 | 10 | 0.1 | 9 | 30 | 77.1 | 76.97 |
| 18 | 20 | 0.3 | 6 | 45 | 87.7 | 88.09 |
| 19 | 30 | 0.5 | 3 | 60 | 92.7 | 93.29 |
| 20 | **10** | **0.5** | **3** | **60** | **100** | **99.48** |
| 21 | 30 | 0.5 | 9 | 30 | 78.7 | 78.59 |
| 22 | 10 | 0.5 | 9 | 60 | 97 | 97.01 |
| 23 | 20 | 0.3 | 6 | 30 | 82.9 | 82.13 |
| 24 | 20 | 0.5 | 6 | 45 | 98.9 | 98.17 |
| 25 | 30 | 0.3 | 6 | 45 | 82 | 81.30 |
| 26 | 10 | 0.3 | 6 | 45 | 89.8 | 89.75 |
| 27 | 10 | 0.5 | 9 | 30 | 87.3 | 87.22 |
| 28 | 10 | 0.1 | 3 | 30 | 79.8 | 79.29 |
| 29 | 20 | 0.3 | 6 | 60 | 89.7 | 89.43 |
| 30 | 30 | 0.1 | 3 | 60 | 79.3 | 78.65 |

- **Photocatalytic Activity**

In the photocatalytic activity of FZT Y@WDS under visible light irradiation, the coated ZrO_2_ and TiO_2_ shells act as mediators for the light-generated holes and electron transfer and prevent the recombination of holes and electrons. The positive effect of Fe_3_O_4_ yolk in the form of Fe^3+^ takes into account the formation of Fe^2+^ species by the transfer of light-generated electrons from TiO_2_ shell to ZrO_2_ shell and then to Fe^3+^. When visible light is irradiated onto the FZT Y@WDS surface, electrons and holes are generated. Fe^3+^ ions can act as electron and hole traps to form Fe^2+^ and Fe^4+^ ions, which are less stable compared to Fe3+ ions. Therefore, it tends to generate Fe^3+^. This leads to the formation of ^•^OH radicals and O_2_ anions. Hydroxyl radicals are considered the main active species during the photocatalytic oxidation reaction. Semiconductor properties, pH values of solutions and phase structure of ZrO_2_ and TiO_2_ shells had a significant effect on the formation of ^•^OH. The rate of formation of ^•^OH on the anatase phase of TiO_2_ and the synthesized materials is much higher than that of ^•^OH in other semiconductors such as rutile TiO_2_. The direct reaction with H_2_O/OH^-^ to produce ^•^OH and the lifetime of electrons and holes in the titanium dioxide shell structure was relatively long.

- **Experimental Design Analysis**

In order to evaluate the effects of process variables on the removal of NAP, experiments were performed using Design-Expert® 7.0.0 software for the FZT Y@WDS sample. After evaluating the experimental results, the quadratic function for photocatalyst removal was determined using Design-Expert. To estimate the polynomial coefficients, least squares regression was used. The effect of the independent parameters on the photocatalytic activity of the synthesized FZT Y@WDS catalysts can be formulated as Eq. (S6):

| Sqrt (NAP Removal) =+6.83932 + [0.051787× NAP Concentration]-[1.44322×C_Cat_.]+ [0.237894×pH] + [0.060409×Time] + [0.015757× NAP Concentration×C_Cat._] - [0.002582× NAP Concentration×pH] - [0.000188× NAP Concentration×Time] - [0.018060× C_Cat._×pH] + [0.002419×C_Cat._×Time] + [0.000387×pH×Time]-[0.001395× NAP Concentration^²^] + [4.75883×C_Cat._^²^] - [0.020437×pH^²^] - [0.000522×Time^²^] | (S6) |
| --- | --- |

The equation in terms of the actual factors can be used to make predictions about the response at given levels of each factor. In this case, the levels should be given in the original units for each factor. This equation should not be used to determine the relative influence of each factor because the coefficients are scaled to correspond to the units of each factor and the intercept is not in the center of the design space. The results of the quadratic responses for degradation in the form of analysis of variance (ANOVA) are shown in Table S4. Experimental Data Statistical analyzes of linear, two-factor, quadratic, and cubic interactive models were performed, and the results are summarized in Table S4.

**Table S4:** Analysis of variance for the obtained responses

| Source | Sum of Squares | df | Mean Square | F-value | p-value |  |
| --- | --- | --- | --- | --- | --- | --- |
| Mean vs Total | 2558.68 | 1 | 2558.68 |  |  |  |
| Linear vs Mean | 4.14 | 4 | 1.03 | 37.97 | < 0.0001 |  |
| 2FI vs Linear | 0.1322 | 6 | 0.0220 | 0.7630 | 0.6078 |  |
| Quadratic vs 2FI | **0.5330** | 4 | **0.1333** | **126.20** | **< 0.0001** | Suggested |
| Cubic vs Quadratic | 0.0072 | 8 | 0.0009 | 0.7311 | 0.6668 | Aliased |
| Residual | 0.0086 | 7 | 0.0012 |  |  |  |
| Total | 2563.50 | 30 | 85.45 |  |  |  |

Choose the highest order polynomial where the additional terms are significant and the model is not biased. The p-values related to the quadratic model were low (0.0001), indicating the significance of the model (Table S4).

The results of the analysis of variance (ANOVA) for the linear model are summarized in Table S5. The model is based on the calculated F value of 324.95 and P value of 1×10^-4^. It is noteworthy. The larger F-value and smaller P-value calculated for catalytic loading (parameter A) compared to the other parameters indicate that this parameter is the most important parameter.

### Table S5: The results of analysis of variance (ANOVA) for the prepared catalysts

| Source | Sum of Squares | df | Mean Square | F-value | p-value |  |
| --- | --- | --- | --- | --- | --- | --- |
| Model | 4.80 | 14 | 0.3431 | 324.92 | < 0.0001 | significant |
| A- NAP Concentration | 0.9748 | 1 | 0.9748 | 923.20 | < 0.0001 |  |
| B-C_Cat._ | 2.15 | 1 | 2.15 | 2035.46 | < 0.0001 |  |
| C-pH | 0.3575 | 1 | 0.3575 | 338.58 | < 0.0001 |  |
| D-Time | 0.6563 | 1 | 0.6563 | 621.59 | < 0.0001 |  |
| AB | 0.0159 | 1 | 0.0159 | 15.05 | 0.0015 |  |
| AC | 0.0960 | 1 | 0.0960 | 90.92 | < 0.0001 |  |
| AD | 0.0128 | 1 | 0.0128 | 12.09 | 0.0034 |  |
| BC | 0.0019 | 1 | 0.0019 | 1.78 | 0.2021 |  |
| BD | 0.0008 | 1 | 0.0008 | 0.7983 | 0.3857 |  |
| CD | 0.0049 | 1 | 0.0049 | 4.60 | 0.0487 |  |
| A² | 0.0505 | 1 | 0.0505 | 47.78 | < 0.0001 |  |
| B² | 0.0939 | 1 | 0.0939 | 88.91 | < 0.0001 |  |
| C² | 0.0877 | 1 | 0.0877 | 83.01 | < 0.0001 |  |
| D² | 0.0357 | 1 | 0.0357 | 33.82 | < 0.0001 |  |
| Residual | 0.0158 | 15 | 0.0011 |  |  |  |
| Lack of Fit | 0.0038 | 10 | 0.0004 | 1.72 | 0.2867 | not significant |
| Pure Error | 0.0016 | 5 | 0.0003 |  |  |  |
| Cor Total | 4.82 | 29 | R² | 0.9967 | | |
| Std. Dev. | 0.0325 | | Adjusted R² | 0.9936 | | |
| Mean | 9.24 | | Predicted R² | 0.9828 | | |
| C.V. % | 0.3519 | | Adeq Precision | 79.2216 | | |

The model F value of 324.92 means that the model is significant. There is only a 0.01% chance that such a large F value could occur due to noise. P-values of less than 0.0500 mean that the model terms are significant. In this case, A, B, C, D, AB, AC, AD, CD, A^2^, B^2^, C^2^, D^2^ are significant model terms. Values greater than 0.1000 indicate that the model terms are not significant. If there are many non-significant model terms (other than those required to support the hierarchy), model reduction may improve your model. The lack-of-fit F-value of 4.57 means that there is a 5.37% chance that such a large lack- of-fit F-value could occur due to noise. A non-significant fitting deficiency is good. we want the model to fit.

- **Accuracy of the Developed Model**

The accuracy of models developed by regression can be checked by determining the R^2^ coefficient, which is a measure of dispersion about the mean. The values of R^2^ and R^2^ adjusted for degradation are 0.9967 and 0.9936, respectively. In addition, the optimum conditions for maximum NAP degradation and minimum catalyst dosage by RSM were determined under the following conditions: Time 60 min, pH equal to 3, NAP 10 mg/L and C_cat._ 0.5 gr/L. Moreover, the degradation under optimal conditions is correlated. Comparison of the real data with the experimental data in Table S3 shows good accuracy. Figure S2 (a) shows that the residuals are randomly distributed, indicating that the proposed models are adequate and there is no reason to suspect that the assumptions of independence or constant variance are violated. Figure S2 (c) shows a plot of the predicted and actual responses. From this plot, it can be seen that the data points are distributed relatively close to the diagonal. Moreover, the coefficient of determination, which is close to one for the degradation response, confirms these results. To validate the proposed model, the correctness of the predicted values should be verified. The comparison between the experimental and predicted values for NAP removal was used to investigate the accuracy of the developed model. Figure S2 (c), (d) shows the linear relationship between the experimental and predicted values for NAP removal, which confirms the validity of the developed model. The distribution of the experimental data and the corresponding predicted values around the line in a narrow range clearly shows the validity of the model.


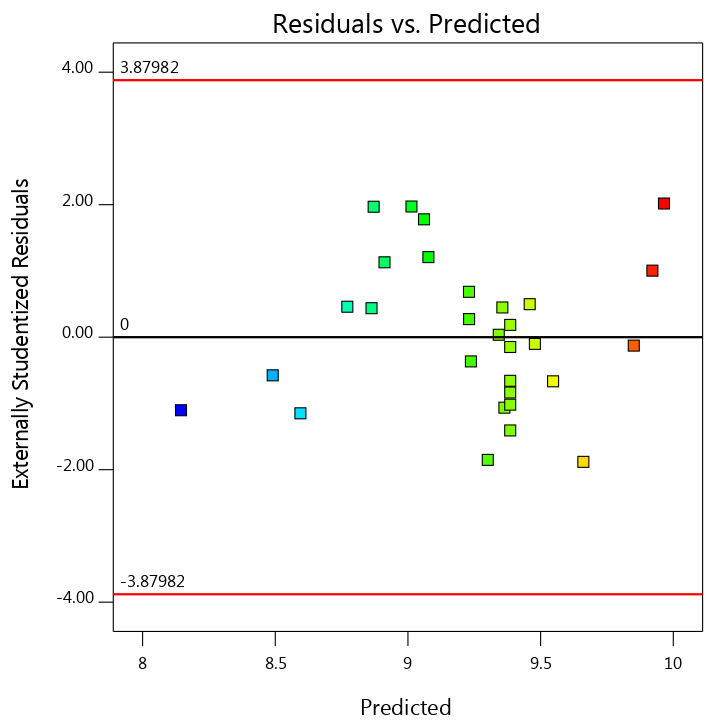

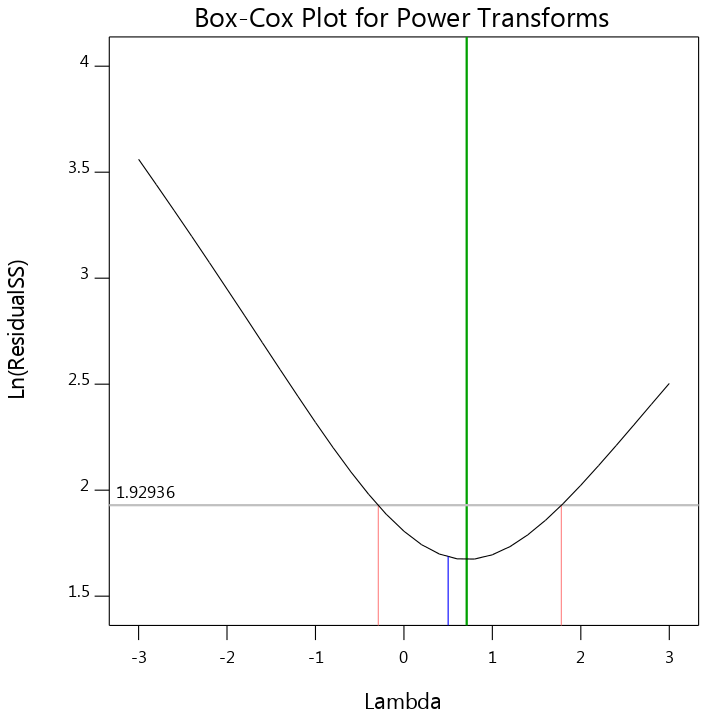


| (a) | (b) |
| --- | --- |


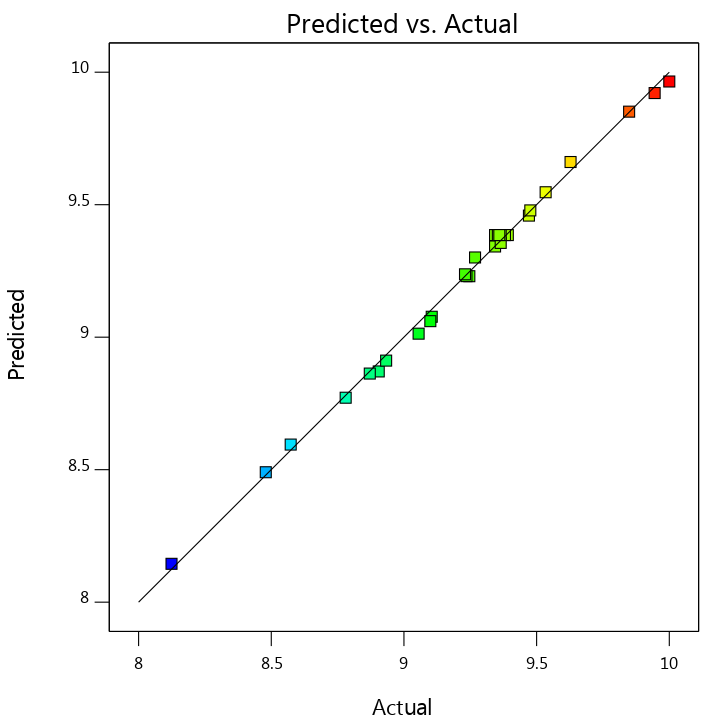

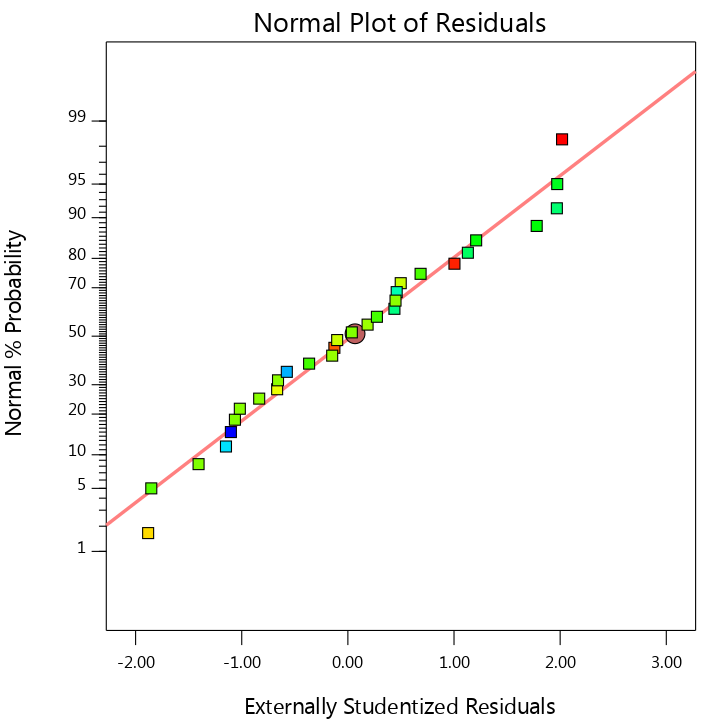


| (c) | (d) |
| --- | --- |

**Fig. S2:** Plot of (a) residual vs. predicted response for degradation (b) predicted responses versus experimental values for degradation, (c) interaction of process variables on degradation at midpoints of variable ranges (d). normal plot residuals

The comparison between all factors at a selected point in the considered design space is represented by a perturbation diagram. This plot for NAP degradation is shown in Figure S3. The degradation response was plotted by varying only one factor in its range while holding the other factors constant. This plot shows the effect of all factors in the middle of the design space (pH=6, irradiation time=45 min, C_cat_=0.3 g/L, NAP =20 ppm). The strong curvature of the time response indicates that the degradation of NAP responds very quickly to this factor, which can also be concluded from Eq. (S1) (i.e., catalyst amount has the highest coefficient among the other variables). The relatively flat pH line showed the least effect of this factor on the degradation of NAP in the design space. The rate of degradation decreased with increasing NAP and pH, which is clear and acceptable.


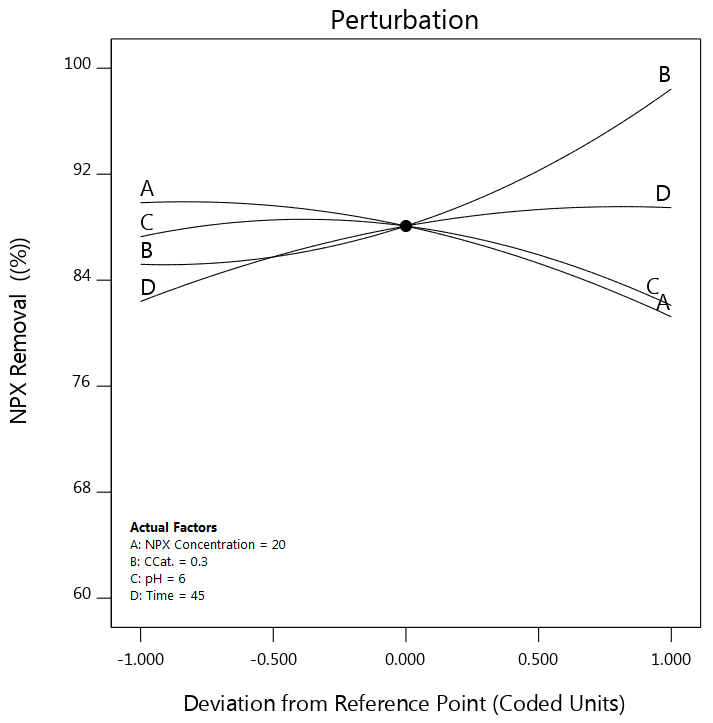


**Fig. S3:** Plot of (a) residual vs. predicted response for degradation (b) predicted responses versus experimental values for degradation, (c) interaction of process variables on degradation at midpoints of variable ranges (d) perturbation at pH=6, Irradiation Time= 45 min, C_cat._=0.3 g/L, C_NAP_= 20 mg/L

- **Effect of Process Parameters on NAP Degradation**

**Solution pH**

the pH plays an important role in the whole process and adsorption capacity, as it affects the surface charge of the adsorbent, the degree of ionization of the substances in the solution, the separation of the functional groups in the active sites and also the chemistry of the solution. At acidic pH, the adsorption capacity is high; at alkaline pH, it tends to decrease. At low pH, the active sites on the surface of the photocatalyst are protonated and the charge density on the surface of the photocatalyst increases. The point at which the positive and negative charges balance is called the isoelectric point, because the isoelectric point is 5.8, i.e., at a pH of 5, the absorbing surface is negatively charged, and at a pH below 5, the charge potential on the absorbing surface is positive, i.e., at this pH it is low. NAP Anions are attracted by electrostatic forces from the positive charges generated on the photocatalyst. At pH above 5, electrostatic repulsion occurs between the negative ions on the absorbing surface, so the repulsive force between NAP and the photocatalyst increases and the removal efficiency decreases. The highest removal rate for the synthesized Fe_3_O_4_, FZ Y@S and FZT Y@WDS photocatalysts at pH 5 is shown in Figure S4 (a) as 88, 94 and 95%, respectively.

**Process Time**

Time is one of the fundamental parameters for the efficiency of the NAP. To investigate the influence of time, the experiment was performed for different periods of time under conditions with a pH of 5, a catalyst concentration of 0.5 g/L of solution, an initial concentration of NAP of 30 mg/L, and a temperature of 25 ℃. The results showed that the amount of removal increases up to 60 min, and after that it does not have much effect on the removal. To justify this phenomenon, it can be said that at the beginning of the reaction, by increasing the contact time, the absorbed particles have more opportunity to advance to the photocatalyst. The amount of absorption of NAP during the contact time of the first 60 min was much higher and faster than the subsequent times. As can be seen in Figure S4 (b), the curve had a steep increase during the first 60 min, and as the contact time increased, the increase of this curve became slower and was finally fixed after 60 min. At this time, after 60 min, the contacts show no significant changes in the amount of NAP absorbed. This may be due to the filling of the pores of the photocatalyst or the difficult access of the NAP molecules to the active sites on the surface of the photocatalyst. However, the rapid absorption of NAP during the initial contact times may be due to the large surface area of the photocatalyst (nanoreactor), which provides many active sites for the NAP molecules.

**Photocatalyst Concentration**

The experiments were performed with a concentration of NAP 30 mg/L at a pH of 5 and a time of 60 min. The results showed that the removal efficiency increased with increasing the amount of photocatalyst, although increasing the concentration above 0.5 g/L did not have much effect on the removal efficiency. This could be due to the increase in the surface area of the photocatalyst or the improved access of NAP molecules to the pores of the photocatalyst. As shown in Figure S4 (c), the removal efficiencies for the synthesized Fe_3_O_4_, FZ Y@S, and FZT Y@WDS photocatalysts are 63, 69, and 78%, respectively, in the amount of 0.2 g/L absorber, and with increasing adsorbent amount of 0.5 g/L, a steep increase in absorption efficiency is shown, but in the amount of 0.5 g/L, the changes in absorption efficiency were almost constant. Therefore, the adsorbent amount of 0.5 g/L is the optimum value for the synthesized Fe_3_O_4_, FZ Y@S and FZT Y@WDS photocatalysts with an absorption efficiency of 84, 91 and 94%, respectively.

**NAP Concentration**

As can be seen from Figure S4 (d), the removal efficiency decreased with increasing NAP concentration for different photocatalyst compositions. The decrease in removal efficiency by increasing the concentration of NAP can be explained by the fact that the amount of photocatalyst, contact time, and pH are the same at all concentrations, so the number of radicals generated is also the same at all concentrations. The second reason for this phenomenon is that as the concentration of NAP increases, the emitted visible light is absorbed by the molecules of NAP and does not reach the surface of all photocatalyst particles; since not all photocatalyst particles are stimulated, the amount of decomposition decreases significantly. Increasing the initial concentration of NAP had a negative effect on the removal efficiency, i.e., as the initial concentration of the pollutant increased, the removal efficiency decreased after reaching the optimal value, which is normal because as the initial concentration of the pollutant increases, its residual amount also increases. Another reason is the saturation of the adsorbent surface at high concentrations of NAP. In general, for a given concentration of the photocatalyst, as the concentration of the pollutant increases, the percentage of absorption and the amount of material absorbed decrease and increase, respectively. At lower initial concentrations of NAP, the ratio between the initial number of molecules of the absorbing substance and the available active sites is low, so the amount of absorption is independent of the initial concentration, but at higher concentrations of the attracting substance, the access to the absorption sites is lower, so the elimination of NAP depends on the initial concentration. The result of the study showed that increasing the initial concentration of NAP has an effect on the absorption capacity method, so when the concentration of NAP was increased from 5 to 35 mg/L, the maximum absorption capacity was reached at a concentration of 10 mg/L. The reason for this is the increase in the driving force caused by the increase in the number of NAP molecules (concentration gradient).

**Fig. S4:** (a). The effect of different pH (initial NAP concentration=30 mg/L, catalyst concentration=0.5 g/L, irradiation time=60 min), (b). The effect of different times (initial NAP concentration=30 mg/L, catalyst concentration=0.5 g/L, pH=5), (c). The effect of different photocatalyst concentration (initial NAP concentration=30 mg/L, irradiation time=60 min, pH=5), (d). The effect of initial pollutant concentrations (catalyst concentration=0.5 g/L, irradiation time=60 min, pH=5) on the NAP removal efficiency.

- **Optimization and Validation of NAP Removal**

Optimization and validation using RSM experimental results showed that complete removal of NAP is possible. Model optimization was performed to achieve optimal conditions under these conditions. To achieve maximum removal of NAP, the independent variables of pH and irradiation time, pollutant concentration of NAP, and catalyst dosage should be set to 5.283, 57.364 min, 13.815 mg/L, and 0.499 g/L, respectively, resulting in 101.953% removal of NAP, as predicted by the model. However, to achieve the highest removal rate of NAP, the developed model shows a wide range of independent parameters as shown in Figure S5. The results show that the complete removal of NAP was achieved under these conditions. The obtained results suggest that the proposed model is valid.


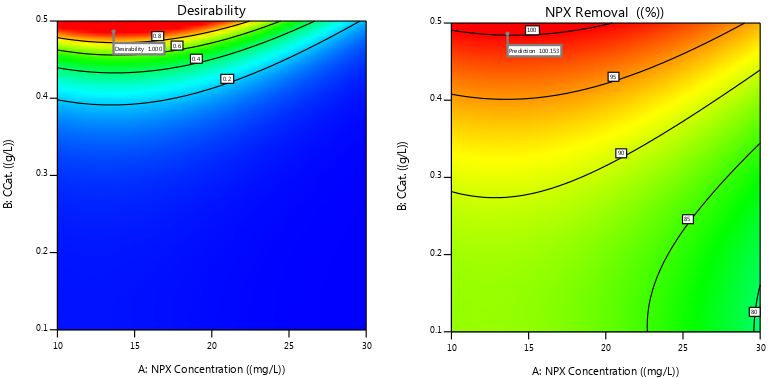


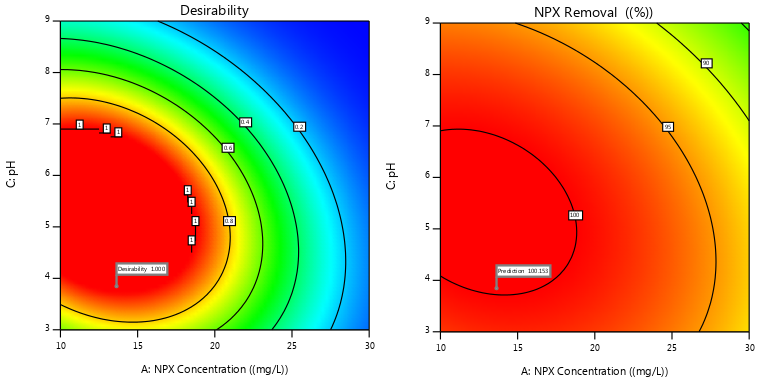


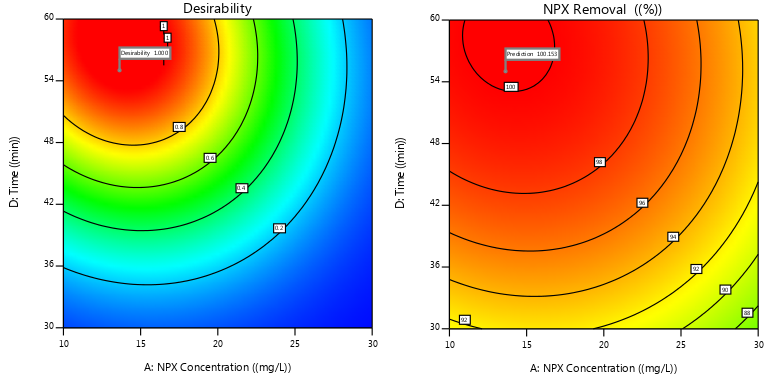


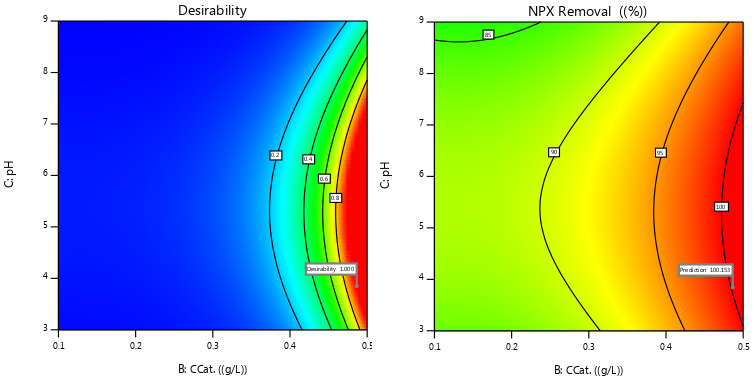


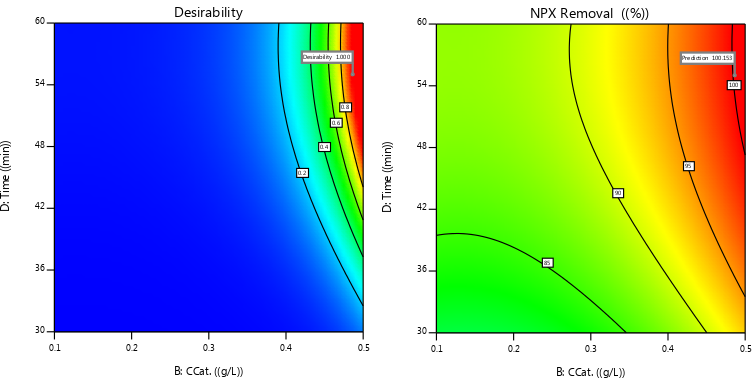


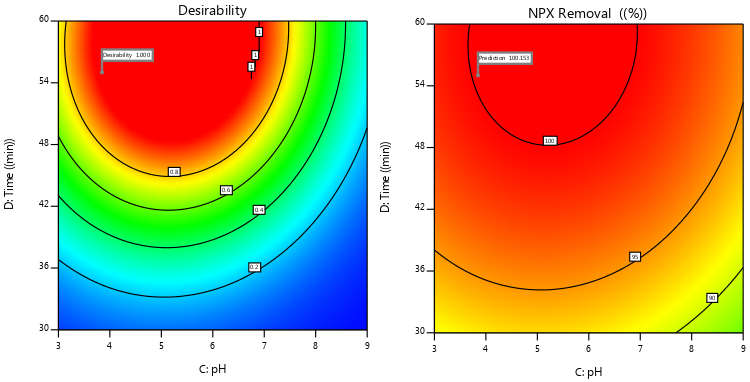


**Fig. S5:** Optimum values of independent variables pH and irradiation time, NAP pollutant concentration and catalyst dose for NAP removal

- **Kinetic Mechanism of NAP Adsorption**

Table S6 shows that the correlation coefficient for the synthesized FZT Y@WDS photocatalysts in the Freundlich and Langmuir models for NAP is 0.9832 and 0.9502, respectively. The kinetic data were analyzed using different kinetic models to determine the kinetic mechanism of NAP adsorption on the surface of the FZT Y@WDS nanoreactor (see Table S6).

**Table S6:** NAP adsorption on the FZT Y@WDS nanoreactor with Equations of adsorption kinetic models

| Model | Equation | Parameter | | Ref |
| --- | --- | --- | --- | --- |
| Elovich | q_t_= (1/β)(Ln(αβ))+(1/β)Ln(t) | R^2^ | 0.9192 | [46-48] |
|  |  | α | 76.178 |  |
|  |  | β | 0.303 |  |
| Freundlich | $\mathrm{Log}Q_{e}=LogK_{f}+\frac{1}{n}C_{e}$ | R^2^ | 0.9832 | [49] |
|  |  | $\mathrm{Log}K_{f}$ | 3.793 |  |
|  |  | $1/n$ | 3.271 |  |
| Langmuir | $\frac{1}{Q_{e}}=\left( \frac{1}{Q_{0}b} \right)\frac{1}{C_{e}}+\frac{1}{Q_{0}}$ | R^2^ | 0.9502 | [50] |
|  |  | $Q_{0}$ | 0.84 |  |
|  |  | b | 2.587 |  |
| Weber and Moris | q_t_ =C+K_int_(t)^1/2^ | R^2^ | 0.9497 | [46-48, 51] |
|  |  | K_int_ | 2.084 |  |
|  |  | C | 10.97 |  |
| Pseudo 2^nd^ order  (McKay-Ho) | (t/q_t_) =1/(K_2_.q_e_^2^)+(1/q_e_).t | R^2^ | 0.9893 | [52, 53] |
|  |  | K_2_ | 0.011 |  |
|  |  | Calculated q_e_ | 27.23 |  |
|  |  | Experimental q_e_ | 26.21 |  |
| Pseudo 1^st^ order  (Lagergeren) | Log (q_e_-q_t_) = Log q_e_-(K_1_/2.303).t | R^2^ | 0.9688 | [52, 54] |
|  |  | K_1_ | 0.069 |  |
|  |  | Calculated q_e_ | 15.93 |  |
| q_e_: FZT Y@WDS nanoreactor adsorbed NAP (mg. g^-1^) at equilibrium.  q_t_: FZT Y@WDS nanoreactor adsorbed NAP (mg. g^-1^) at determined time interval (t).  K_1_: Pseudo-1^st^ order adsorption rate constant (1/min).  K_2_: Pseudo-2^nd^ order adsorption rate constant (g/mg.min)  α: initial adsorption rate (mg. g^-1^.min^-1^).  β: the desorption constant (g. mg^-1^)  K_int_: Intra-particle diffusion rate constant (mg.g^-1^.min-^1/2^).  C: Thickness of the boundary | | | | |

The Elovich model yielded a regression coefficient R^2^ of 0.9192, indicating lower linearity. In contrast, the correlation coefficient for the kinetic model of Langmuir-Hinshelwood (L-H) was 0.9977 for NAP and was higher than all other adsorption kinetics models. The calculated qe value was found to be 27.23 mg/g for NAP, which is very close to the experimental values (see Table S6).

As shown in Table S7, the photocatalyst synthesized in this study degrades NAP more efficiently than previous catalysts. This study showed that the three types of NPs have a synergistic effect on photocatalysis in ternary nanoreactors.

**Table S7:** Evaluation of the NAP removal efficiency in the presence of synthesized photocatalysts

| Type of Photocatalyst | Organic Pollutant | NAP Concentration  (mg/L) | Irradiation Time  (min) | Catalyst Concentration  (g/L) | pH | Degradation Efficiency  (%) | Reference |
| --- | --- | --- | --- | --- | --- | --- | --- |
| N-doped TiO_2_/SiO_2_/Fe_3_O_4_ | NAP | 9.33 | 217.08 | 0.06 | 4.29 | 96.32 | [8] |
| Fe_3_O_4_/MWCNTs | NAP | 10 | - | 0.4 | 7 | 83 | [9] |
| ZnO | NAP | 40 | 120 | 0.5 | 7 | 98.7 | [10] |
| FeCo2O4@g-C3N4 | NAP | 50 | 180 | 5 | 4.2 | 91 | [11] |
| HTNM | NAP | 0.5 | 180 | 1.5 | 7 | 99.9 | [12] |
| P25-TiO_2_/TEOS | NAP | 5 | 600 | 0.003 | 6 | 94 | [13] |
| MoS_2_-CeO_2_-ZrO_2_ | NAP | 11.51 | 40 | 0.5 | 5.8 | 96 | [14] |
| TiO_2_ | NAP | 0.184 | 120 | 0.1 | 6.15 | 40 | [15] |
| FZT Y@WDS | **NAP** | **10** | **60** | **0.5** | **3** | **100** | **Present Work** |

​Table S8 compares the pollution degradation of photocatalysts with different yolk@shell structures. The use of visible light to activate the present synthesized photocatalyst to remove organic pollutants from wastewater in the shortest time is one of the most important features of the synthesized yolk@shell structure.

**Table S8:** Summary of the catalytic performance of nanoreactors applied in photocatalytic degradation of pollutants applications**.**

| **Degradation of Pollutants**  𝑅+𝑂𝐻−→𝑅−+𝐻_2_𝑂 | **Particle name/**  **Structure*** | **Illumination** | **Pollutant Degradation Efficiency** | **Tested Time** | **Ref** |
| --- | --- | --- | --- | --- | --- |
|  | Au@r-GO/TiO_2_  Y@S | Visible light  (λ=400 nm) | RhB = 99% | 100 min | [16] |
|  | Fe_3_O_4_@C@TiO_2_  Y@S | UV light | RhB ≈ 99% | 90 min | [17] |
|  | C@TiO_2_  Y@S | UV light  (λ=365 nm) | RhB ≈ 99% | 30 min | [18] |
|  | Fe_3_O_4_@TiO_2_@Ag  Y@S | UV and visible lights | AMP = 98.7% (UV)  AMP=91.5% (Visible) | 360 min | [19] |
|  | **FZT Y@WDS**  **Y@S** | **Visible light**  **(λ=400 nm)** | **NAP=100%** | **60 min** | **Present Work** |

**Reference**

1. Mullet, M., et al., *A simple and accurate determination of the point of zero charge of ceramic membranes.* Desalination, 1999. **121**(1): p. 41-48.

2. Meites, L., *Handbook of analytical chemistry.* Soil Science, 1963. **96**(5): p. 358.

3. Yu, J., et al., *Bifunctionality from synergy: CoP nanoparticles embedded in amorphous CoOx nanoplates with heterostructures for highly efficient water electrolysis.* Advanced Science, 2018. **5**(9): p. 1800514.

4. Sawyer, D.T., A. Sobkowiak, and J.L. Roberts, *Electrochemistry for chemists*. 1995: Wiley.

5. Friis, E.P., et al., *Dynamics of Pseudomonas aeruginosa azurin and its Cys3Ser mutant at single-crystal gold surfaces investigated by cyclic voltammetry and atomic force microscopy.* Electrochimica Acta, 1998. **43**(9): p. 1114-1122.

6. Bard, A.J. and L.R. Faulkner, *Fundamentals and applications.* Electrochemical methods, 2001. **2**(482): p. 580-632.

7. Zoski, C.G., *Handbook of electrochemistry*. 2006: Elsevier.

8. Amini, Z., et al., *Synthesis of N-doped TiO2/SiO2/Fe3O4 magnetic nanocomposites as a novel purple LED illumination-driven photocatalyst for photocatalytic and photoelectrocatalytic degradation of naproxen: optimization and different scavenger agents study.* Journal of Environmental Science and Health, Part A, 2019. **54**(12): p. 1254-1267.

9. Huaccallo-Aguilar, Y., et al., *Naproxen removal by CWPO with Fe3O4/multi-walled carbon nanotubes in a fixed-bed reactor.* Journal of Environmental Chemical Engineering, 2021. **9**(2): p. 105110.

10. Sabouni, R. and H. Gomaa, *Photocatalytic degradation of pharmaceutical micro-pollutants using ZnO.* Environ Sci Pollut Res Int, 2019. **26**(6): p. 5372-5380.

11. Palanivel, B., et al., *Activation of Persulfate for Improved Naproxen Degradation Using FeCo2O4@g-C3N4 Heterojunction Photocatalysts.* ACS Omega, 2021. **6**(50): p. 34563-34571.

12. Fan, G., et al., *Photocatalytic degradation of naproxen by a H2O2-modified titanate nanomaterial under visible light irradiation.* Catalysis Science & Technology, 2019. **9**(17): p. 4614-4628.

13. Zhang, H., et al., *Photocatalytic degradation of four non-steroidal anti-inflammatory drugs in water under visible light by P25-TiO2/tetraethyl orthosilicate film and determination via ultra performance liquid chromatography electrospray tandem mass spectrometry.* Chemical Engineering Journal, 2015. **262**: p. 1108-1115.

14. Talukdar, K., et al., *Rational construction of CeO2–ZrO2@MoS2 hybrid nanoflowers for enhanced sonophotocatalytic degradation of naproxen: Mechanisms and degradation pathways.* Composites Part B: Engineering, 2021. **215**: p. 108780.

15. Méndez-Arriaga, F., J. Gimenez, and S. Esplugas, *Photolysis and TiO2 Photocatalytic Treatment of Naproxen: Degradation, Mineralization, Intermediates and Toxicity.* Journal of Advanced Oxidation Technologies, 2008. **11**(3): p. 435-444.

16. Wang, M., et al., *Yolk@Shell Nanoarchitecture of Au@r-GO/TiO2 Hybrids as Powerful Visible Light Photocatalysts.* Langmuir, 2015. **31**(22): p. 6220-6228.

17. Chen, L., et al., *A novel strategy to fabricate multifunctional Fe3O4@C@TiO2 yolk–shell structures as magnetically recyclable photocatalysts.* Nanoscale, 2014. **6**(12): p. 6603-6608.

18. Joo, J.B., et al., *Tailored synthesis of C@TiO2 yolk–shell nanostructures for highly efficient photocatalysis.* Catalysis Today, 2016. **264**: p. 261-269.

19. Zhao, Y., et al., *Controlled synthesis and photocatalysis of sea urchin-like Fe3O4@TiO2@Ag nanocomposites.* Nanoscale, 2016. **8**(9): p. 5313-5326.
